# Supplementary figures and images for: Noradrenergic deficits contribute to apathy in Parkinson’s disease through the precision of expected outcomes
Source: PLoS Comput Biol. 2022 May 9;18(5):e1010079. doi: 10.1371/journal.pcbi.1010079 (PMC9119485; doi:10.1371/journal.pcbi.1010079)

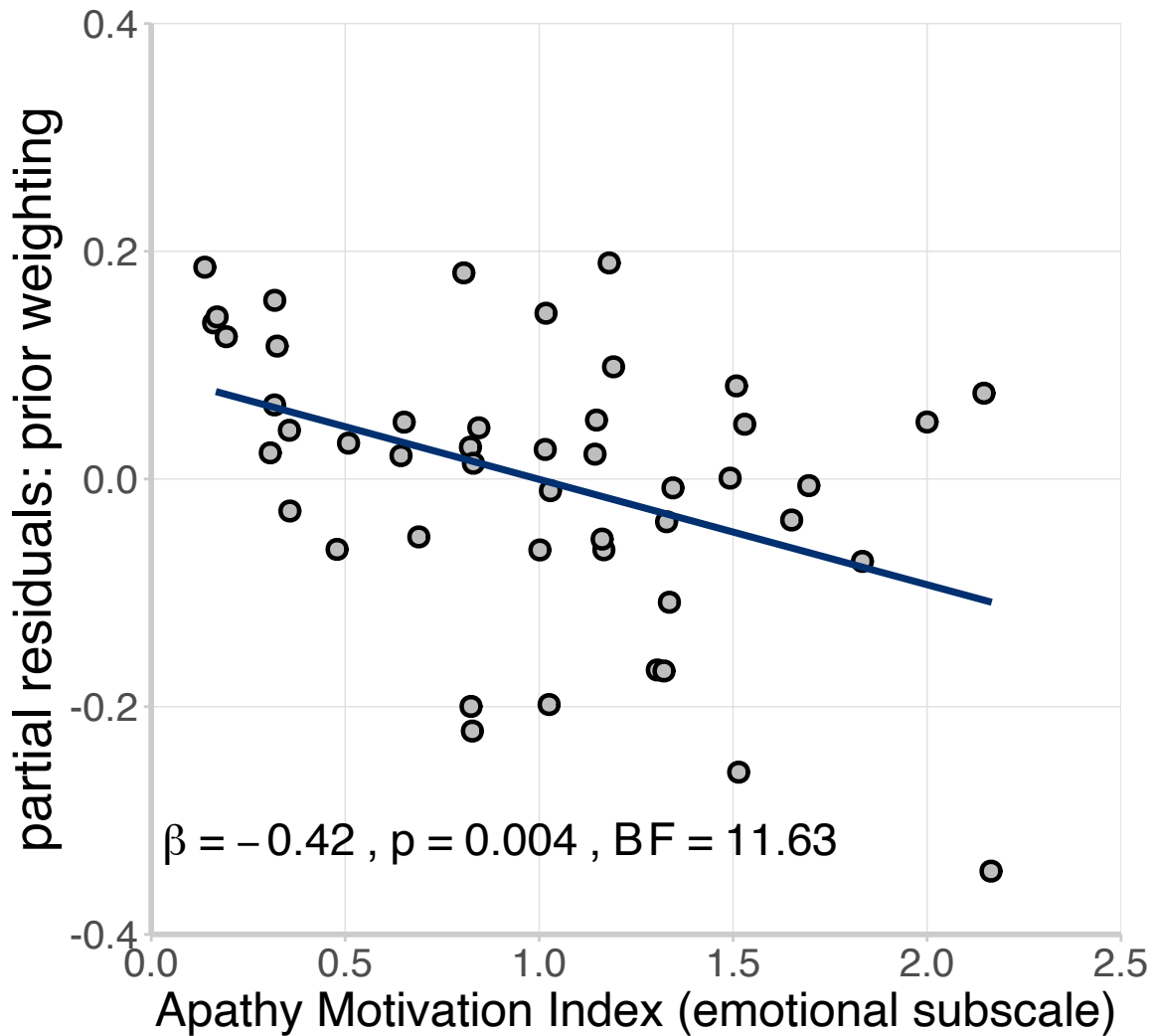

Supplement: S1 Fig — Data from Hezemans et al. [43], demonstrating the relationship between trait apathy (measured using the Apathy Motivation Index) and prior weighting, adjusted for task performance variability (i.e., partial residuals). Note that observations with identical questionnaire scores were horizontally jittered to avoid overlaid dots. Full statistics for the regression coefficient of apathy: β = -0.42, SE = 0.14, t(44) = -3.02, p = .004; BF = 11.63. (PDF) [file pcbi.1010079.s001.pdf]

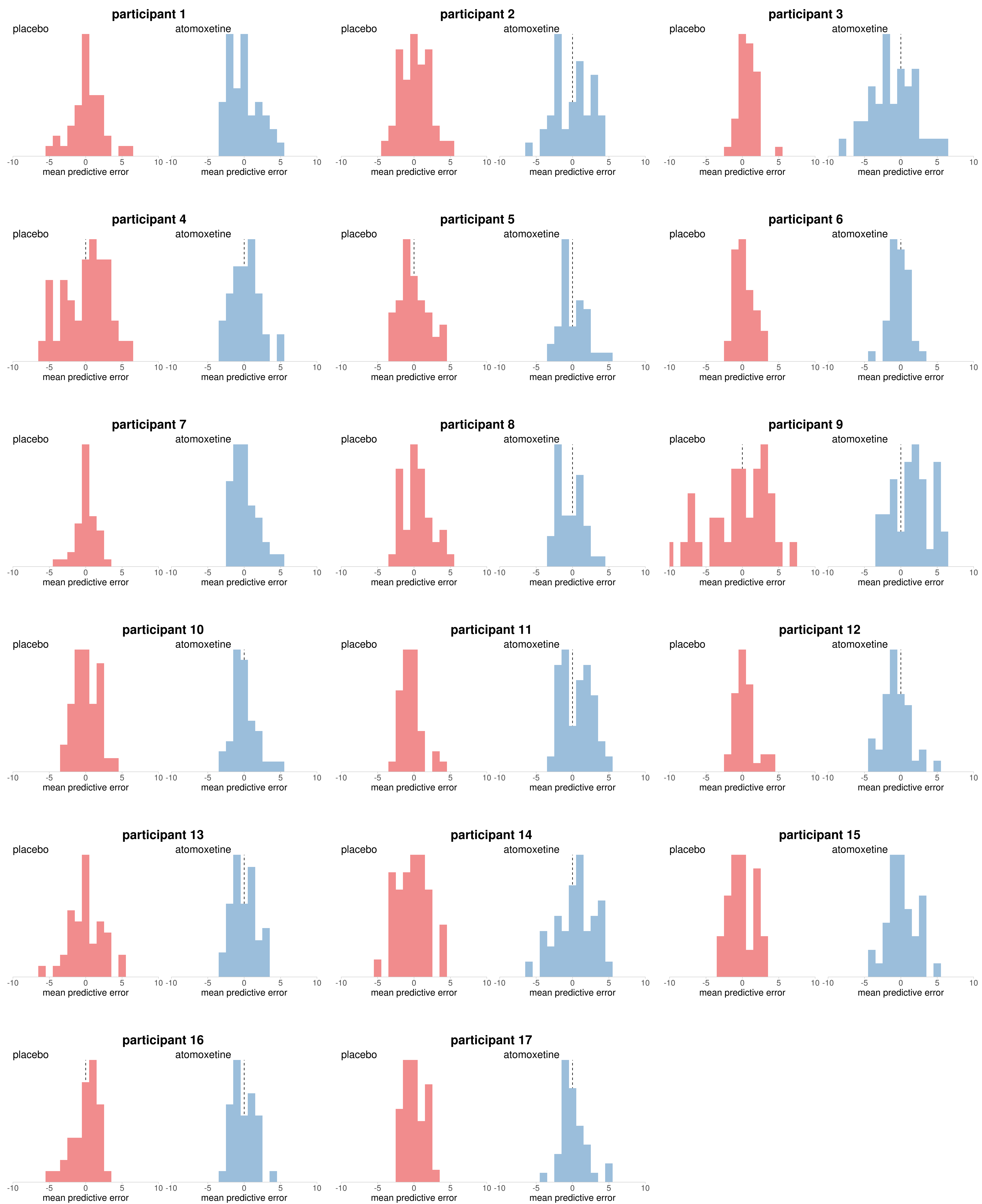

Supplement: S5 Fig — Each panel illustrates the distribution of the mean predictive error of the model–that is, the observed responses minus simulated responses drawn from the model’s posterior predictive distribution, averaged across Markov Chain Monte Carlo samples. These histograms can therefore be interpreted as the distributions of residuals. (PDF) [file pcbi.1010079.s005.pdf]
