# Supplementary material for: Noradrenergic deficits contribute to apathy in Parkinson’s disease through the precision of expected outcomes
Source: PLoS Comput Biol. 2022 May 9;18(5):e1010079. doi: 10.1371/journal.pcbi.1010079 (PMC9119485; doi:10.1371/journal.pcbi.1010079)
Supplement: S2 Fig — Participant-level parameters were sampled from latent group-level distributions. We allowed for atomoxetine-induced changes in the standard deviations of the prior and sensory evidence distributions. Session-level parameters were then used to obtain trial-level posterior distributions of the final ball position. The observed estimates of performance were modelled as samples from the trial-level posterior distributions. The model is represented in plate notation: shaded nodes represent observed data whereas white nodes represent latent variables; rectangular nodes represent discrete or fixed variables whereas circular nodes represent continuous variables; and double-bordered white nodes represent deterministic variables whereas single-bordered white nodes represent stochastic variables. (PDF) [file pcbi.1010079.s002.pdf]

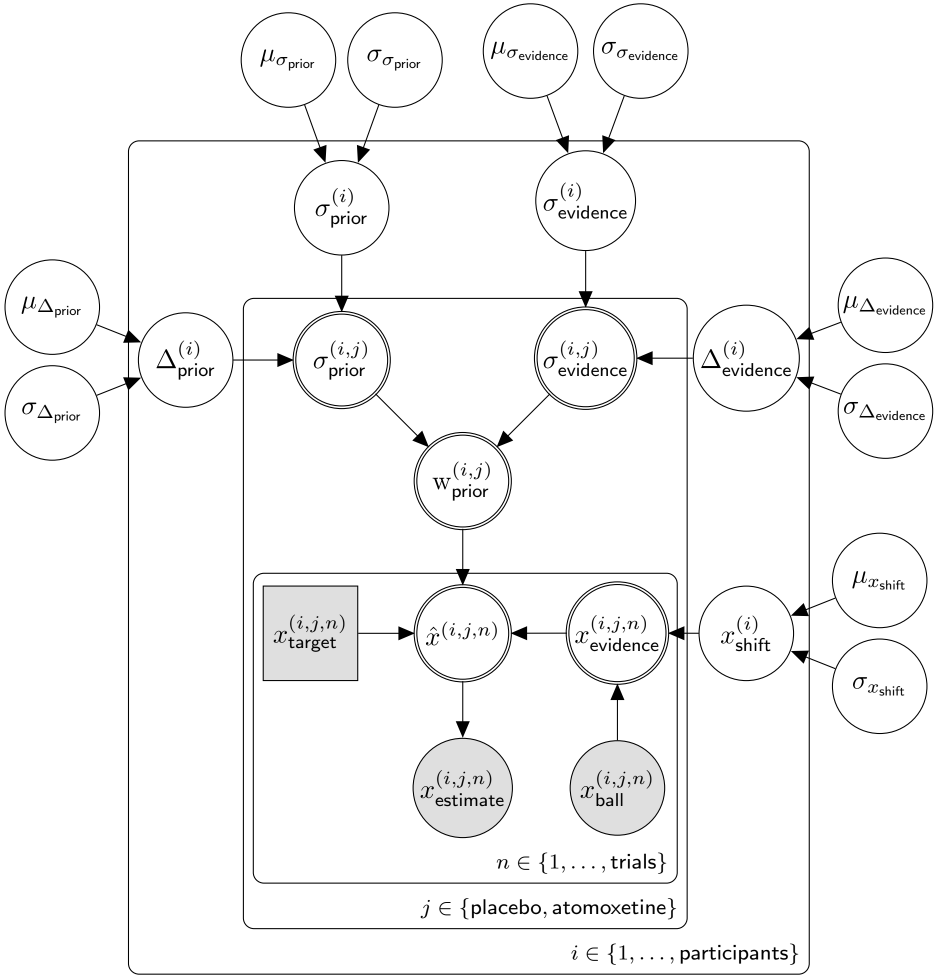

group-level prior SD:

$$\mu_{\sigma_{\text{prior}}} \sim \mathcal{N}(100, 10) \qquad \mu_{\sigma_{\text{prior}}} > 0$$
$$\sigma_{\sigma_{\text{prior}}} \sim \text{Cauchy}(0, 5) \qquad \sigma_{\sigma_{\text{prior}}} > 0$$

group-level drug effect on prior SD:

$$\mu_{\Delta_{\text{prior}}} \sim \mathcal{N}(0, 10)$$
$$\sigma_{\Delta_{\text{prior}}} \sim \text{Cauchy}(0, 5) \qquad \sigma_{\Delta_{\text{prior}}} > 0$$

group-level sensory evidence SD:

$$\mu_{\sigma_{\text{evidence}}} \sim \mathcal{N}(100, 10) \qquad \mu_{\sigma_{\text{evidence}}} > 0$$
$$\sigma_{\sigma_{\text{evidence}}} \sim \text{Cauchy}(0, 5) \qquad \sigma_{\sigma_{\text{evidence}}} > 0$$

group-level drug effect on sensory evidence SD:

$$\mu_{\Delta_{\text{evidence}}} \sim \mathcal{N}(0, 10)$$
$$\sigma_{\Delta_{\text{evidence}}} \sim \text{Cauchy}(0, 5) \qquad \sigma_{\Delta_{\text{evidence}}} > 0$$

group-level sensory evidence shift:

$$\mu_{x_{\text{shift}}} \sim \mathcal{N}(0, 10)$$
$$\sigma_{x_{\text{shift}}} \sim \text{Cauchy}(0, 5) \qquad \sigma_{x_{\text{shift}}} > 0$$

---

participant-level prior SD:

$$\sigma^{(i)}_{\text{prior}} \sim \mathcal{N}\left(\mu_{\sigma_{\text{prior}}}, \sigma_{\sigma_{\text{prior}}}\right) \qquad \sigma^{(i)}_{\text{prior}} > 0$$

participant-level drug effect on prior SD:

$$\Delta^{(i)}_{\text{prior}} \sim \mathcal{N}\left(\mu_{\Delta_{\text{prior}}}, \sigma_{\Delta_{\text{prior}}}\right)$$

participant-level sensory evidence SD:

$$\sigma^{(i)}_{\text{evidence}} \sim \mathcal{N}\left(\mu_{\sigma_{\text{evidence}}}, \sigma_{\sigma_{\text{evidence}}}\right) \qquad \sigma^{(i)}_{\text{evidence}} > 0$$

participant-level drug effect on sensory evidence SD:

$$\Delta^{(i)}_{\text{evidence}} \sim \mathcal{N}\left(\mu_{\Delta_{\text{evidence}}}, \sigma_{\Delta_{\text{evidence}}}\right)$$

participant-level sensory evidence shift:

$$x^{(i)}_{\text{shift}} \sim \mathcal{N}\left(\mu_{x_{\text{shift}}}, \sigma_{x_{\text{shift}}}\right)$$

---

session-level prior SD:

$$\sigma^{(i,j)}_{\text{prior}} \leftarrow \begin{cases} \sigma^{(i)}_{\text{prior}}, & \text{if } j = \text{placebo} \\ \sigma^{(i)}_{\text{prior}} + \Delta^{(i)}_{\text{prior}}, & \text{if } j = \text{atomoxetine} \end{cases}$$

session-level sensory evidence SD:

$$\sigma^{(i,j)}_{\text{evidence}} \leftarrow \begin{cases} \sigma^{(i)}_{\text{evidence}}, & \text{if } j = \text{placebo} \\ \sigma^{(i)}_{\text{evidence}} + \Delta^{(i)}_{\text{evidence}}, & \text{if } j = \text{atomoxetine} \end{cases}$$

session-level prior weighting:

$$\text{w}^{(i,j)}_{\text{prior}} \leftarrow \frac{\left[\sigma^{(i,j)}_{\text{evidence}}\right]^2}{\left[\sigma^{(i,j)}_{\text{evidence}}\right]^2 + \left[\sigma^{(i,j)}_{\text{prior}}\right]^2}$$

session-level posterior SD:

$$\sigma^{(i,j)}_{\hat{x}} \leftarrow \sqrt{\frac{\left[\sigma^{(i,j)}_{\text{evidence}}\right]^2 \cdot \left[\sigma^{(i,j)}_{\text{prior}}\right]^2}{\left[\sigma^{(i,j)}_{\text{evidence}}\right]^2 + \left[\sigma^{(i,j)}_{\text{prior}}\right]^2}}$$

---

trial-level sensory evidence mean:

$$x^{(i,j,n)}_{\text{evidence}} \leftarrow x^{(i,j,n)}_{\text{ball}} + x^{(i)}_{\text{shift}}$$

trial-level posterior mean:

$$\hat{x}^{(i,j,n)} \leftarrow \text{w}^{(i,j)}_{\text{prior}} \cdot x^{(i,j,n)}_{\text{target}} + \left[1 - \text{w}^{(i,j)}_{\text{prior}}\right] \cdot x^{(i,j,n)}_{\text{evidence}}$$

trial-level estimate of performance:

$$x^{(i,j,n)}_{\text{estimate}} \sim \mathcal{N}\left(\hat{x}^{(i,j,n)}, \sigma^{(i,j)}_{\hat{x}}\right)$$
