# Supplementary material for: Noradrenergic deficits contribute to apathy in Parkinson’s disease through the precision of expected outcomes
Source: PLoS Comput Biol. 2022 May 9;18(5):e1010079. doi: 10.1371/journal.pcbi.1010079 (PMC9119485; doi:10.1371/journal.pcbi.1010079)
Supplement: S3 Fig — In addition to the model presented in the manuscript, we fit three variants of the model with restrictions on the number of drug-induced change parameters. For each model variant, we computed the leave-one-out information criterion (LOOIC) and the widely applicable information criterion (WAIC) as estimates of the model’s expected predictive accuracy. For both measures, the ‘full’ model which included drug-induced change parameters for both the prior and sensory evidence standard deviations was strongly preferred over the three more restricted model variants. (PDF) [file pcbi.1010079.s003.pdf]

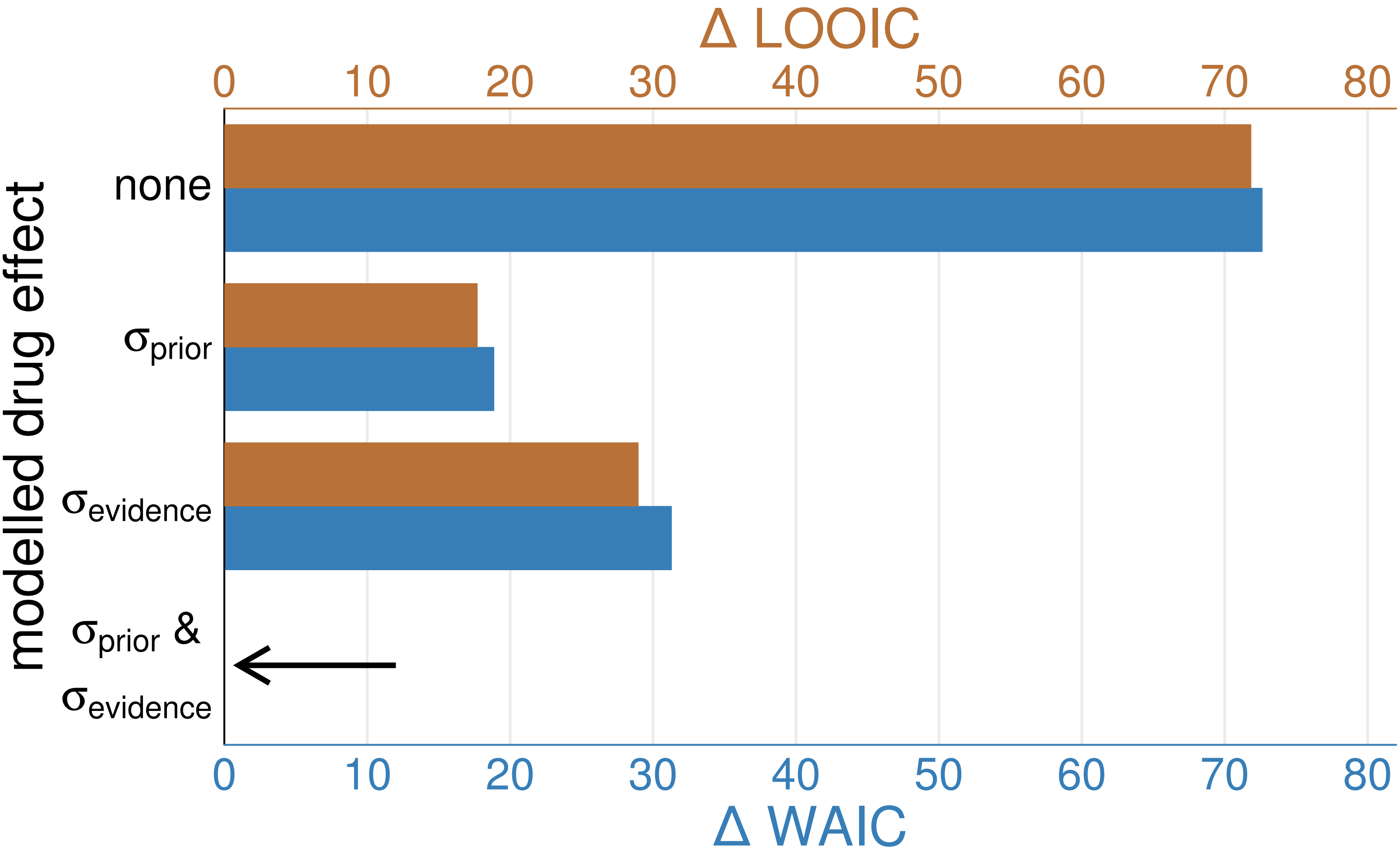

Δ LOOIC
0
10
20
30
40
50
60
70
80
modelled drug effect
none
σprior
σevidence
σprior &
σevidence
0
10
20
30
40
50
60
70
80
Δ WAIC
