# Supplementary material for: Noradrenergic deficits contribute to apathy in Parkinson’s disease through the precision of expected outcomes
Source: PLoS Comput Biol. 2022 May 9;18(5):e1010079. doi: 10.1371/journal.pcbi.1010079 (PMC9119485; doi:10.1371/journal.pcbi.1010079)
Supplement: S4 Fig — Each panel compares the observed responses (light-coloured density plot and histogram) to distributions of simulated responses drawn from the model’s posterior predictive distribution (dark-coloured density traces). (PDF) [file pcbi.1010079.s004.pdf]

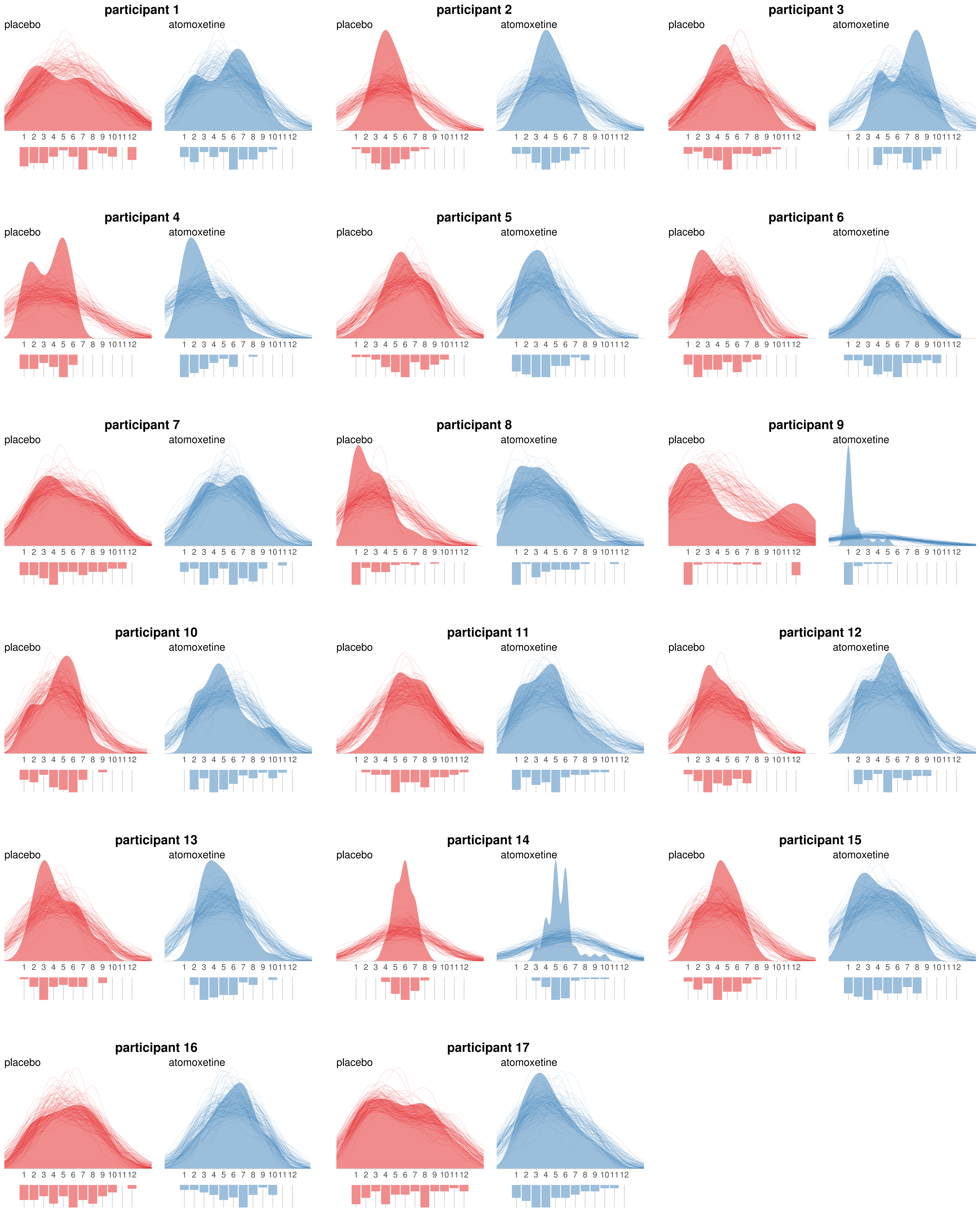
participant 1
placebo
atomoxetine
participant 2
placebo
atomoxetine
participant 3
placebo
atomoxetine
participant 4
placebo
atomoxetine
participant 5
placebo
atomoxetine
participant 6
placebo
atomoxetine
participant 7
placebo
atomoxetine
participant 8
placebo
atomoxetine
participant 9
placebo
atomoxetine
participant 10
placebo
atomoxetine
participant 11
placebo
atomoxetine
participant 12
placebo
atomoxetine
participant 13
placebo
atomoxetine
participant 14
placebo
atomoxetine
participant 15
placebo
atomoxetine
participant 16
placebo
atomoxetine
participant 17
placebo
atomoxetine
1 2 3 4 5 6 7 8 9 10 11 12
