# Supplementary material for: Noradrenergic deficits contribute to apathy in Parkinson’s disease through the precision of expected outcomes
Source: PLoS Comput Biol. 2022 May 9;18(5):e1010079. doi: 10.1371/journal.pcbi.1010079 (PMC9119485; doi:10.1371/journal.pcbi.1010079)
Supplement: S1 Text — Table A. Descriptive statistics and group comparisons of questionnaires. Data are presented as mean (SD). Group comparisons were performed with independent samples t-tests. The stated p-values are uncorrected; none survived p < .05 after correction for multiple comparisons. Abbreviations: BF, Bayes Factor for the alternative hypothesis over the null hypothesis, where > 3 would indicate positive evidence in favour of a group difference; BIS, Barratt Impulsiveness Scale; HADS, Hospital Anxiety and Depression Scale; MEI, Motivation and Energy Inventory; CAARS, Conners’ Adult ADHD Rating Scale; RBDSQ, REM sleep Behaviour Disorder Screening Questionnaire; CBI-R, Cambridge Behavioural Inventory–Revised. Fig A. Density plots of questionnaire scores for participants with Parkinson’s disease (blue) and controls (orange). The questionnaire scores were z-scored to bring the different questionnaires onto a common scale (note that this transformation does not affect group comparisons for a given questionnaire outcome). Tick marks reflect individual data points. Abbreviations: BIS, Barratt Impulsiveness Scale; MEI, Motivation and Energy Inventory; HADS, Hospital Anxiety and Depression Scale; CAARS, Conners’ Adult ADHD Rating Scale. (DOCX) [file pcbi.1010079.s006.docx]

**S1 Text: Mood and behaviour questionnaires**

All participants completed the following set of self-report questionnaires that assessed mood and various behavioural symptoms: Apathy Scale [1], Barratt Impulsiveness Scale [2], Motivation and Energy Inventory [3], Hospital Anxiety and Depression Scale [4], and Conners’ Adult ADHD Rating Scale [5]. Individuals with Parkinson’s disease additionally completed the REM sleep behaviour disorder screening questionnaire [6]. Furthermore, relatives or friends of the Parkinson’s disease patients completed informant-rated versions of the Conners’ Adult ADHD Rating Scale and the Apathy Scale, as well as a general mood and behavioural symptom inventory (Cambridge Behavioural Inventory Revised)[7].

**Table A | Descriptive statistics and group comparisons of questionnaires.**

| **Measure** |  | **PD** | **Controls** | ***BF*** | ***p*** |
| --- | --- | --- | --- | --- | --- |
| Apathy Scale | Total Score (self-rated) | 12.68 (5.77) | 10.58 (5.09) | 0.58 | .212 |
|  | Total Score (informant-rated) | 13.13 (5.59) |  |  |  |
| BIS | Total Score | 56.45 (10.34) | 56.15 (9.67) | 0.3 | .924 |
|  | Attention | 14.16 (4.3) | 14.23 (3.72) | 0.3 | .953 |
|  | Motor | 20.08 (2.65) | 20.85 (3.38) | 0.39 | .398 |
|  | Non-planning | 22.21 (5.4) | 21.08 (4.44) | 0.38 | .459 |
| HADS | Anxiety | 4.53 (3.2) | 4.31 (3.53) | 0.3 | .83 |
|  | Depression | 3.95 (2.68) | 2.88 (2.76) | 0.58 | .202 |
| MEI | Total Score | 98.05 (21.3) | 108.96 (16.71) | 1.29 | .073 |
|  | Mental | 44.11 (8.97) | 47.35 (8.09) | 0.57 | .22 |
|  | Physical | 23.95 (6.95) | 29.35 (5.91) | 6.18 | .01 |
|  | Social | 30 (7.34) | 32.27 (5.31) | 0.53 | .261 |
| CAARS (self-rated) | Inattention / Memory Problems | 5.42 (3.58) | 4.19 (3.06) | 0.55 | .235 |
|  | Hyperactivity / Restlessness | 3.11 (2.71) | 2.77 (2.05) | 0.33 | .652 |
|  | Impulsivity / Emotional Lability | 1.84 (1.5) | 2.77 (2.05) | 0.9 | .087 |
|  | Problems with Self-Concept | 2.26 (2.33) | 3.88 (4.12) | 0.77 | .102 |
|  | ADHD Index | 6.79 (4.26) | 8.38 (4.51) | 0.53 | .233 |
| CAARS (observer-rated) | Inattention / Memory Problems | 4.24 (2.51) |  |  |  |
|  | Hyperactivity / Restlessness | 1.58 (1.92) |  |  |  |
|  | Impulsivity / Emotional Lability | 1.21 (1.23) |  |  |  |
|  | Problems with Self-Concept | 2.32 (2.11) |  |  |  |
|  | ADHD Index | 4.47 (3.42) |  |  |  |
| RBDSQ |  | 4.58 (3.45) |  |  |  |
| CBI-R | Total Score | 15.13 (13.6) |  |  |  |
|  | Abnormal Behaviour | 0.84 (1.12) |  |  |  |
|  | Beliefs | 0.37 (1.21) |  |  |  |
|  | Eating Habits | 0.95 (1.58) |  |  |  |
|  | Everyday Skills | 1.16 (2.41) |  |  |  |
|  | Memory and Orientation | 4.66 (3.9) |  |  |  |
|  | Mood | 1.26 (2.1) |  |  |  |
|  | Motivation | 2.26 (3.35) |  |  |  |
|  | Stereotypic and Motor Behaviours | 0.79 (1.4) |  |  |  |
|  | Self Care | 0.42 (0.84) |  |  |  |
|  | Sleep | 2.42 (2.17) |  |  |  |

*Note*: Data are presented as mean (SD). Group comparisons were performed with independent samples t-tests. The stated *p*-values are uncorrected; none survived *p* < .05 after correction for multiple comparisons. Abbreviations: BF, Bayes Factor for the alternative hypothesis over the null hypothesis, where > 3 would indicate positive evidence in favour of a group difference; BIS, Barratt Impulsiveness Scale; HADS, Hospital Anxiety and Depression Scale; MEI, Motivation and Energy Inventory; CAARS, Conners’ Adult ADHD Rating Scale; RBDSQ, REM sleep Behaviour Disorder Screening Questionnaire; CBI-R, Cambridge Behavioural Inventory – Revised.


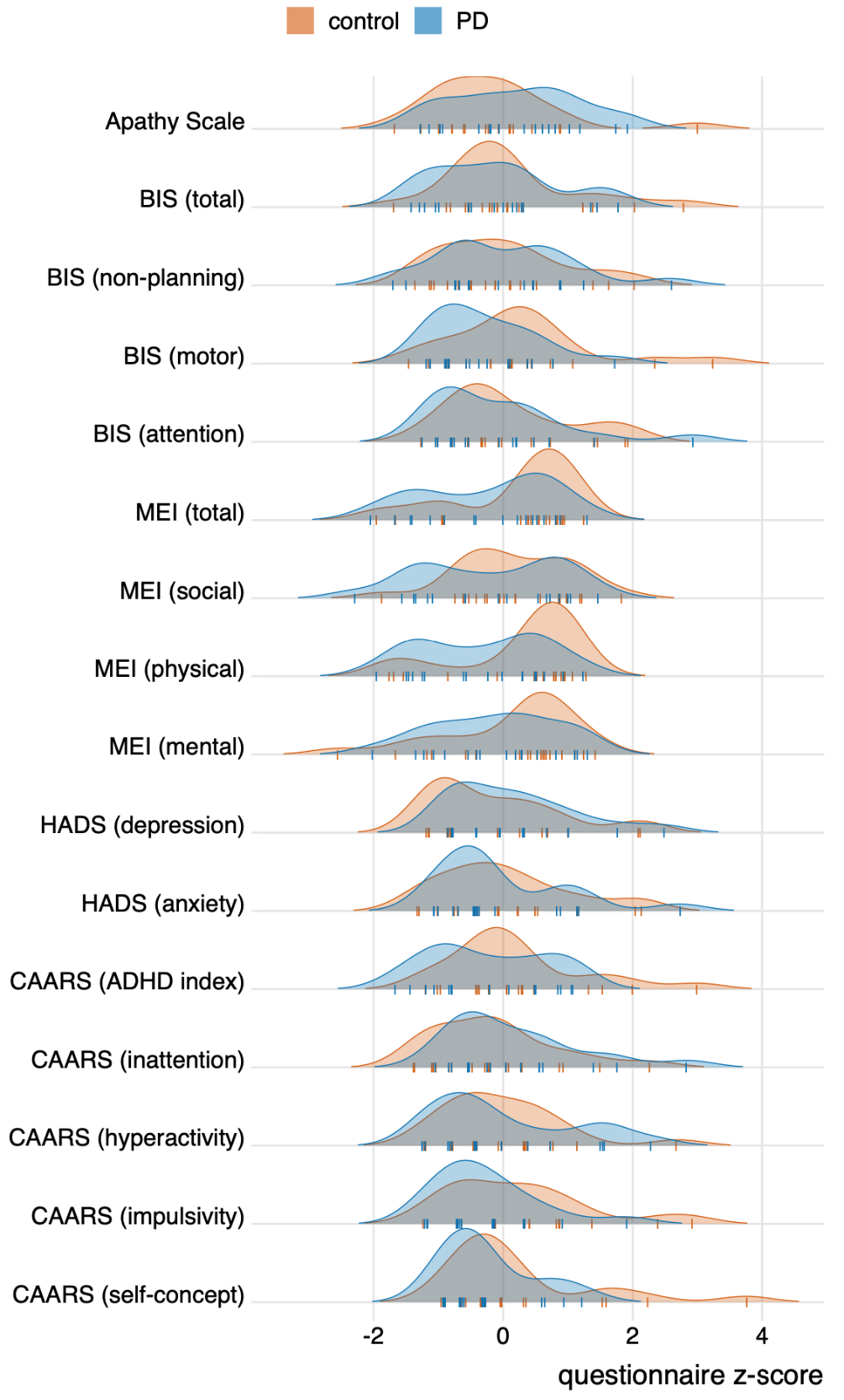


**Fig A | Density plots of questionnaire scores for participants with Parkinson’s disease (blue) and controls (orange).** The questionnaire scores were z-scored to bring the different questionnaires onto a common scale (note that this transformation does not affect group comparisons for a given questionnaire outcome). Tick marks reflect individual data points. Abbreviations: BIS, Barratt Impulsiveness Scale; MEI, Motivation and Energy Inventory; HADS, Hospital Anxiety and Depression Scale; CAARS, Conners’ Adult ADHD Rating Scale.

**References**

1. Starkstein SE, Mayberg HS, Preziosi TJ, Andrezejewski P, Leiguarda R, Robinson RG. Reliability, validity, and clinical correlates of apathy in Parkinson’s disease. J Neuropsychiatry Clin Neurosci. 1992;4: 134–139. doi:10.1176/jnp.4.2.134

2. Patton JH, Stanford MS, Barratt ES. Factor structure of the barratt impulsiveness scale. J Clin Psychol. 1995;51: 768–774. doi:10.1002/1097-4679(199511)51:6\textless768::AID-JCLP2270510607\textgreater3.0.CO;2-1

3. Fehnel SE, Bann CM, Hogue SL, Kwong WJ, Mahajan SS. The development and psychometric evaluation of the motivation and energy inventory (MEI). Qual Life Res. 2004;13: 1321–1336. doi:10.1023/B:QURE.0000037502.64077.4d

4. Zigmond AS, Snaith RP. The Hospital Anxiety and Depression Scale. Acta Psychiatr Scand. 1983;67: 361–370. doi:10.1111/j.1600-0447.1983.tb09716.x

5. Conners CK, Erhardt D, Epstein JN, Parker JDA, Sitarenios G, Sparrow E. Self-ratings of ADHD symptoms in adults I: Factor structure and normative data. J Atten Disord. 1999;3: 141–151. doi:10.1177/108705479900300303

6. Stiasny‐Kolster K, Mayer G, Schäfer S, Möller JC, Heinzel‐Gutenbrunner M, Oertel WH. The REM sleep behavior disorder screening questionnaire—A new diagnostic instrument. Mov Disord. 2007;22: 2386–2393. doi:10.1002/mds.21740

7. Wear HJ, Wedderburn CJ, Mioshi E, Williams-Gray CH, Mason SL, Barker RA, et al. The Cambridge Behavioural Inventory revised. Dement Neuropsychol. 2008;2: 102–107. doi:10.1590/S1980-57642009DN20200005
