# Supplementary material for: Noradrenergic deficits contribute to apathy in Parkinson’s disease through the precision of expected outcomes
Source: PLoS Comput Biol. 2022 May 9;18(5):e1010079. doi: 10.1371/journal.pcbi.1010079 (PMC9119485; doi:10.1371/journal.pcbi.1010079)
Supplement: S2 Text — Table A. Backward elimination of fixed effects in the linear mixed effects model predicting prior weighting. Values for predictors are standardised regression coefficients (ß). *p < .05. Drug, atomoxetine vs. placebo condition; LC CNR, Locus Coeruleus Contrast to Noise Ratio; ICV, total intracranial volume; Ato plasma, atomoxetine plasma concentration; LEDD, Levodopa Equivalent Daily Dose; Session, first vs. second session; UPDRS III, Unified Parkinson’s Disease Rating Scale, motor examination; AIC, Akaike Information Criterion; BIC, Bayesian Information Criterion; Δ AIC / BIC, difference in AIC / BIC with respect to the lowest AIC / BIC value. All models included a fixed effect of the interquartile range of performance error as a covariate of no interest, and a random effect of participants on the intercept. Total intracranial volume was estimated from the T1-weighted MP2RAGE images using the mri_segstats–etiv-only procedure in FreeSurfer v6.0.0. Table B. Bayes Factors for the inclusion of fixed effects in the linear mixed effects model predicting prior weighting. P(incl): prior inclusion probability, i.e. the summed prior probability of models that include the predictor. A priori, all possible restrictions of the full model were deemed to be equally likely (i.e., a uniform prior was assigned to the model space). Thus, P(incl) reflects the proportion of alternative models that included the predictor. P(incl|data): posterior inclusion probability, i.e. the summed posterior probability of models that include the predictor. P(excl|data): posterior exclusion probability, i.e. the summed posterior probability of models that exclude the predictor. BFinclusion: Inclusion Bayes Factor, i.e. the change from prior to posterior inclusion odds. This indicates how much more likely the data are under models that include the predictor, compared to models that exclude the predictor. This analysis was performed using “matched” models, which means that (i) models were not permitted [file pcbi.1010079.s007.docx]

**S2 Text: Role of covariates in the drug × LC CNR interaction on prior weighting**

**Table A | Backward elimination of fixed effects in the linear mixed effects model predicting prior weighting.**

|  | | **Model selection step** | | | | | | | | | | | | | | | |
| --- | --- | --- | --- | --- | --- | --- | --- | --- | --- | --- | --- | --- | --- | --- | --- | --- | --- |
| **Predictors** | | 1 | | | 2 | | | 3 | | | 4 | | | 5 | | | 6 |
| Drug | -2.02 × 10^-3^ | | | -2.02 × 10^-3^ | | | -2.06 × 10^-3^ | | | -2.06 × 10^-3^ | | | -2.06 × 10^-3^ | | | -2.06 × 10^-3^ | |
| LC CNR | -0.02 | | | -0.02 | | | -0.02 | | | -0.06 | | | -0.08 | | | -0.13 | |
| Drug × LC CNR | -0.37^*^ | | | -0.37^*^ | | | -0.38^*^ | | | -0.38^*^ | | | -0.38^*^ | | | -0.38^*^ | |
| ICV | 0.37 | | | 0.37 | | | 0.37 | | | 0.37 | | | 0.35 | | | 0.37^*^ | |
| Age | 0.22 | | | 0.23 | | | 0.23 | | | 0.19 | | | 0.18 | | | **^-^** | |
| Ato plasma | -0.13 | | | -0.13 | | | -0.13 | | | -0.11 | | | - | | | - | |
| LEDD | 0.13 | | | 0.14 | | | 0.14 | | | - | | | - | | | - | |
| Session | 0.07 | | | 0.07 | | | - | | | - | | | - | | | - | |
| UPDRS III | 0.01 | | | - | | | - | | | - | | | - | | | - | |
|  | |  |  | | |  | | |  | | |  | | |  | | |
| **Information Criteria** | | |  | | |  | | |  | | |  | | |  | | |
| AIC | | 100.59 | | | 98.59 | | | 96.89 | | | 95.77 | | | 94.38 | | | 93.69 |
| ∆ AIC | | 6.89 | | | 4.90 | | | 3.20 | | | 2.07 | | | 0.68 | | | 0 |
| BIC | | 120.43 | | | 116.91 | | | 113.68 | | | 111.03 | | | 108.12 | | | 105.91 |
| ∆ BIC | | 14.52 | | | 11.00 | | | 7.78 | | | 5.12 | | | 2.21 | | | 0 |

*Note*. Values for predictors are standardised regression coefficients (ß). ^*^*p* < .05. Drug, atomoxetine vs. placebo condition; LC CNR, Locus Coeruleus Contrast to Noise Ratio; ICV, total intracranial volume; Ato plasma, atomoxetine plasma concentration; LEDD, Levodopa Equivalent Daily Dose; Session, first vs. second session; UPDRS III, Unified Parkinson’s Disease Rating Scale, motor examination; AIC, Akaike Information Criterion; BIC, Bayesian Information Criterion; ∆ AIC / BIC, difference in AIC / BIC with respect to the lowest AIC / BIC value. All models included a fixed effect of the interquartile range of performance error as a covariate of no interest, and a random effect of participants on the intercept. Total intracranial volume was estimated from the T1-weighted MP2RAGE images using the mri_segstats –etiv-only procedure in FreeSurfer v6.0.0 [1].

**Table B | Bayes Factors for the inclusion of fixed effects in the linear mixed effects model predicting prior weighting.**

| **Predictors** | P(incl) | P(incl\|data) | P(excl\|data) | BF_inclusion_ |
| --- | --- | --- | --- | --- |
| Drug | 0.40 | 0.13 | 0.40 | 0.32 |
| LC CNR | 0.40 | 0.19 | 0.33 | 0.59 |
| Drug × LC CNR | 0.20 | 0.48 | 0.05 | 10.06 |
| ICV | 0.50 | 0.58 | 0.42 | 1.40 |
| Age | 0.50 | 0.42 | 0.58 | 0.72 |
| Ato plasma | 0.50 | 0.38 | 0.62 | 0.62 |
| LEDD | 0.50 | 0.38 | 0.62 | 0.61 |
| Session | 0.50 | 0.30 | 0.70 | 0.43 |
| UPDRS III | 0.50 | 0.40 | 0.60 | 0.67 |

*Note.* P(incl): prior inclusion probability, i.e. the summed prior probability of models that include the predictor. A priori, all possible restrictions of the full model were deemed to be equally likely (i.e., a uniform prior was assigned to the model space). Thus, P(incl) reflects the proportion of alternative models that included the predictor. P(incl|data): posterior inclusion probability, i.e. the summed posterior probability of models that include the predictor. P(excl|data): posterior exclusion probability, i.e. the summed posterior probability of models that exclude the predictor. BF_inclusion_: Inclusion Bayes Factor, i.e. the change from prior to posterior inclusion odds. This indicates how much more likely the data are under models that include the predictor, compared to models that exclude the predictor [2]. This analysis was performed using “matched” models, which means that (i) models were not permitted to include an interaction effect without its constituent main effects, and (ii) inclusion probabilities for an interaction effect were based only on the subset of models that contained (at least) the constituent main effects of the interaction [3]. All models included a fixed effect of the interquartile range of performance error as a covariate of no interest, and a random effect of participants on the intercept.**References**

1. Malone IB, Leung KK, Clegg S, Barnes J, Whitwell JL, Ashburner J, et al. Accurate automatic estimation of total intracranial volume: A nuisance variable with less nuisance. NeuroImage. 2015;104: 366–372. doi:10.1016/j.neuroimage.2014.09.034

2. Hinne M, Gronau QF, van den Bergh D, Wagenmakers E-J. A Conceptual Introduction to Bayesian Model Averaging. Adv Methods Pract Psychol Sci. 2020; 2515245919898657. doi:10.1177/2515245919898657

3. Mathôt S. Bayes like a Baws: Interpreting Bayesian Repeated Measures in JASP // Cogsci. 2017. Available: https://www.cogsci.nl/blog/interpreting-bayesian-repeated-measures-in-jasp
