# Supplementary material for: Noradrenergic deficits contribute to apathy in Parkinson’s disease through the precision of expected outcomes
Source: PLoS Comput Biol. 2022 May 9;18(5):e1010079. doi: 10.1371/journal.pcbi.1010079 (PMC9119485; doi:10.1371/journal.pcbi.1010079)
Supplement: S3 Text — Fig A. Decomposing the noradrenergic effects on prior weighting using a plausible values analysis approach. (A-B) Distributions of plausible correlations between the drug effect on prior weighting and the estimated drug effect on the standard deviation of the prior (A) or sensory evidence (B). (C) Distribution of the difference between the plausible correlations. (DOCX) [file pcbi.1010079.s008.docx]

**S3 Text: Decomposing drug effects on prior weighting: plausible values analysis**

In the main manuscript, we examined whether the effect of atomoxetine on prior weighting was differentially associated with the effects of atomoxetine on prior precision and sensory evidence precision. The drug effects on prior and sensory evidence precision were estimated using hierarchical Bayesian estimation of a model of perceptual inference. Hierarchical techniques fit the data from all participants simultaneously, while explicitly accounting for individual differences. Compared to fitting each participant separately, hierarchical modelling produces participant-level parameter estimates that are – on average – closer to the true parameter values, due to shrinkage towards the mean [1]. Although this shrinkage is advantageous in the context of parameter estimation, it can be problematic for post-hoc statistical testing. Specifically, because participant-level parameter estimates from hierarchical models are shrunk towards each other, statistical analyses that pool across these parameter estimates will underestimate the pooled standard deviation, leading to inflated effect sizes and test statistics [2].

To address this issue, we performed a plausible values analysis that accounts for the uncertainty of the participant-level parameter estimates from the hierarchical model [3]. This approach repeats the statistical test of interest for each of the Markov Chain Monte Carlo (MCMC) samples from the joint posterior distribution of the hierarchical model. In other words, the statistical test is repeated for each set of plausible values of the parameters, rather than relying on a single summary of the parameters (e.g., posterior medians). This yields a full posterior distribution of the test statistic, which explicitly represents the estimation uncertainty.

We obtained the distributions of plausible correlations between the drug effect on prior weighting and the estimated drug effects on prior precision (panel A in Fig A) and sensory evidence precision (panel B in Fig A). There was a strongly negative correlation between the drug effect on prior weighting and the drug effect on the standard deviation of the prior, with the distribution of plausible correlations reliably shifted below zero (median = -0.55, 95% QI: [-0.76, -0.27], P(*r* < 0) = 99.98%). In contrast, the distribution of plausible correlations between the drug effect on prior weighting and the drug effect on the standard deviation of sensory evidence was approximately centred on zero (median = -0.04, 95% QI: [-0.37, 0.34], P(*r* < 0) = 57.73%). To examine whether these correlations were different from each other, we calculated the difference between the plausible correlation estimates for each MCMC sample. The distribution of the difference between correlations was clearly shifted below zero (panel C in Fig A; median = -0.51, 95% QI: [-0.93, -0.09], P(∆ *r* < 0) = 99.18%), indicating that the correlation with the drug effect on prior precision was significantly stronger (i.e., more negative) than the correlation with the drug effect on sensory evidence precision.

Taken together, the results from this supplementary analysis are consistent with the results presented in the main manuscript, suggesting that the atomoxetine-induced change in prior weighting was primarily explained by changes in prior precision, and not by changes in sensory evidence precision.


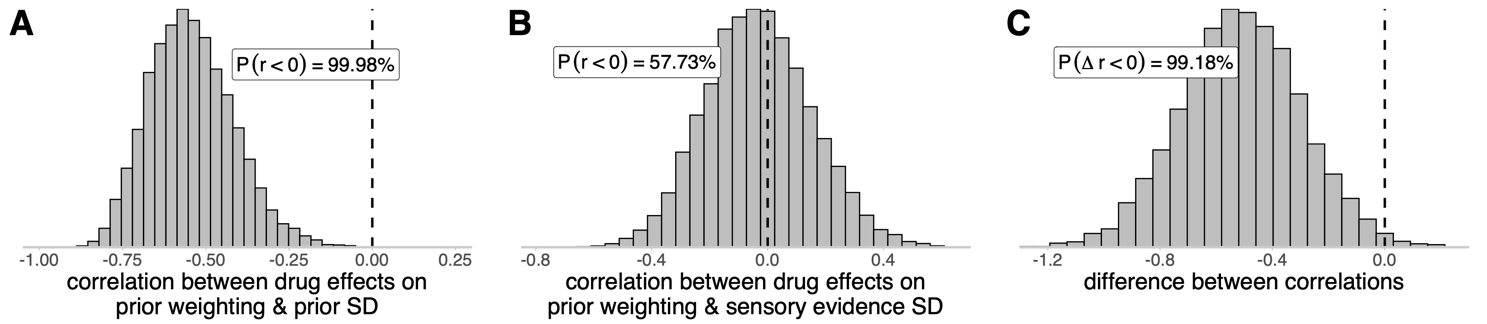


**Fig A | Decomposing the noradrenergic effects on prior weighting using a plausible values analysis approach.** (A-B) Distributions of plausible correlations between the drug effect on prior weighting and the estimated drug effect on the standard deviation of the prior (A) or sensory evidence (B). (C) Distribution of the difference between the plausible correlations.

**References**

1. Farrell S, Lewandowsky S. Computational Modeling of Cognition and Behavior. Cambridge University Press; 2018. Available: https://doi.org/10.1017/CBO9781316272503

2. Boehm U, Marsman M, Matzke D, Wagenmakers E-J. On the importance of avoiding shortcuts in applying cognitive models to hierarchical data. Behav Res Methods. 2018;50: 1614–1631. doi:10.3758/s13428-018-1054-3

3. Ly A, Boehm U, Heathcote A, Turner BM, Forstmann B, Marsman M, et al. A Flexible and Efficient Hierarchical Bayesian Approach to the Exploration of Individual Differences in Cognitive-model-based Neuroscience. In: Moustafa AA, editor. Computational Models of Brain and Behavior. Chichester, UK: John Wiley & Sons, Ltd; 2017. pp. 467–479. doi:10.1002/9781119159193.ch34
