# Supplementary material for: Noradrenergic deficits contribute to apathy in Parkinson’s disease through the precision of expected outcomes
Source: PLoS Comput Biol. 2022 May 9;18(5):e1010079. doi: 10.1371/journal.pcbi.1010079 (PMC9119485; doi:10.1371/journal.pcbi.1010079)
Supplement: S4 Text — Fig A. Task accuracy (median force error) plotted as a function of effort, reward, group, and drug. Dots represent individual participants, and boxplots represent the marginal distribution for a given condition. For the Parkinson’s disease group, the grey lines indicate within-subject change in median force error from placebo to atomoxetine. Box-plot elements: centre line, median; box limits, first and third quartiles; whiskers, most extreme observations within 1.5 × interquartile range from the box limits. Fig B. Task variability (interquartile range of force error) plotted as a function of effort, reward, group, and drug. Dots represent individual participants, and boxplots represent the marginal distribution for a given condition. For the Parkinson’s disease group, the grey lines indicate within-subject change in the interquartile range of force error from placebo to atomoxetine. Box-plot elements: centre line, median; box limits, first and third quartiles; whiskers, most extreme observations within 1.5 × interquartile range from the box limits. Fig C. Prior weighting plotted as a function of effort, reward, group, and drug. Dots represent individual participants, and boxplots represent the marginal distribution for a given condition. For the Parkinson’s disease group, the grey lines indicate within-subject change in prior weighting from placebo to atomoxetine. Box-plot elements: centre line, median; box limits, first and third quartiles; whiskers, most extreme observations within 1.5 × interquartile range from the box limits. Table A. Estimated marginal means of median force error by group, effort, and reward. Table B. Estimated marginal means of median force error by drug, effort, and reward. Table C. Estimated marginal means of interquartile range of force error by group, effort, and reward. Table D. Estimated marginal means of interquartile range of force error by drug, effort, and reward. Table E. Estimated marginal means of prior weighting by group, effort, [file pcbi.1010079.s009.docx]

**S4 Text: Effects of effort and reward on task performance and prior weighting**

The visuomotor task featured experimental manipulations of effort and reward in a 2 × 2 factorial design (low effort vs. high effort; no reward vs. reward). Although the effort and reward factors were not of primary interest for the current study, we nevertheless hereby report the effects of these manipulations on basic task performance and prior weighting.

*Basic task performance*
Basic task performance was assessed with the force error. This was defined as the difference between the observed force response and the force required to stop the ball perfectly on target, expressed as a percentage of the participant’s maximum force (see Materials and Methods for details). For each participant and each condition, we calculated the median force error and interquartile range (IQR) of force error as summary measures of accuracy and variability, respectively. These were separately entered as dependent variables in ANOVAs, with effort and reward as within-subjects factors, and group (controls vs. Parkinson’s disease on placebo) as the between-subjects factor. To test for potential interaction effects between atomoxetine and the experimental manipulations, we ran additional ANOVAs specifically on the Parkinson’s disease group with effort, reward and drug (atomoxetine vs. placebo) as within-subjects factors.

Across PD patients on placebo and controls, median force error was more negative in the high effort condition compared to the low effort condition (Fig A, Table A; *F*_(1, 35)_ = 46.17, *p* < .001; *BF*_inclusion_ = 6.01 × 10^10^), reflecting a tendency to ‘undershoot’ the target when more effort was required. There was no effect of reward on median force error (*F*_(1, 35)_ = 0.001, *p* = .974; *BF*_inclusion_ = 0.17), nor an interaction effect between effort and reward (*F*_(1, 35)_ = 0.57, *p* = .457; *BF*_inclusion_ = 0.27). In terms of performance variability, the interquartile range of force error increased in the high effort condition compared to the low effort condition (Fig B, Table C; *F*_(1, 35)_ = 58.50, *p* < .001; *BF*_inclusion_ = 1.13 × 10^11^). There was no main effect of reward (*F*_(1, 35)_ = 0.005, *p* = .944; *BF*_inclusion_ = 0.18) nor an interaction effect between effort and reward (*F*_(1, 35)_ = 0.57, *p* = .456; *BF*_inclusion_ = 0.26) on the interquartile range of force error. There was no evidence for interaction effects between group, effort and / or reward on either the median force error or interquartile range of force error (all *p*’s > .050; all *BF*_inclusion_ < 1.00). Among the Parkinson’s disease patients, there was no evidence for interaction effects between drug, effort and / or reward on either the median force error or interquartile range of force error (Table B, Table D; all *p*’s > .050; all *BF*_inclusion_ < 1.00).

*Prior weighting*
We estimated prior weighting as the negative of the regression coefficient of performance errors on estimation errors. This analysis was implemented as a linear mixed effects model with a random effect of participants on the slope (see Materials and Methods for details). To estimate prior weighting for each experimental condition, we used a linear mixed effects model with an additional random effect term, that allowed the intercept and slope to vary for each combination of effort and reward within each participant. In other words, this model estimated prior weighting for each participant, as well as adjustments to prior weighting by effort and reward, nested within each participant. These estimates of prior weighting were then entered as the dependent variable in an ANOVA with effort and reward as within-subjects factors, group (controls vs. Parkinson’s disease on placebo) as the between-subjects factor, and performance variability (interquartile range of performance errors) as a between-subjects covariate of no interest. A separate ANOVA was performed specifically on the Parkinson’s disease group with effort, reward, and drug as within-subjects factors and performance variability as a between-subjects covariate of no interest, to explore potential interaction effects between atomoxetine and the experimental manipulations.

Across PD patients on placebo and controls, prior weighting was higher in the high effort condition compared to the low effort condition (Fig C, Table E; *F*_(1, 34)_ = 6.91, *p* = .013; *BF*_inclusion_ = 3.50). There was no effect of reward on prior weighting (*F*_(1, 34)_ = 0.07, *p* = .791; *BF*_inclusion_ = 0.19), nor an interaction effect between effort and reward (*F*_(1, 34)_ = 0.08, *p* = .774; *BF*_inclusion_ = 0.25). There was no evidence for interaction effects between group, effort and / or reward on prior weighting (all *p*’s > .050; all *BF*_inclusion_ < 1.00). Among the Parkinson’s disease patients, there was no evidence for interaction effects between drug, effort and / or reward on prior weighting (Table F; all *p*’s > .050; all *BF*_inclusion_ < 1.00).


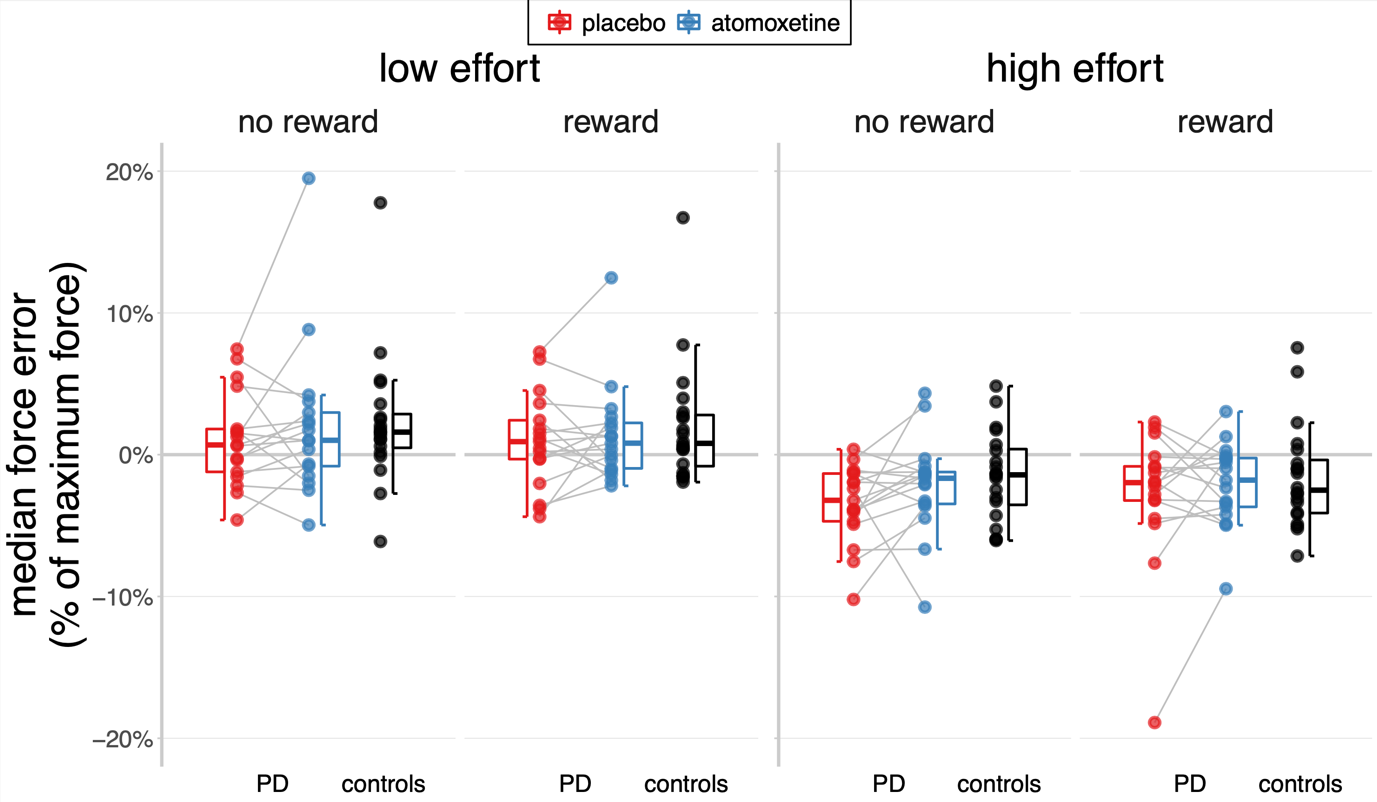


**Fig A | Task accuracy (median force error) plotted as a function of effort, reward, group, and drug.** Dots represent individual participants, and boxplots represent the marginal distribution for a given condition. For the Parkinson’s disease group, the grey lines indicate within-subject change in median force error from placebo to atomoxetine. Box-plot elements: centre line, median; box limits, first and third quartiles; whiskers, most extreme observations within 1.5 × interquartile range from the box limits.


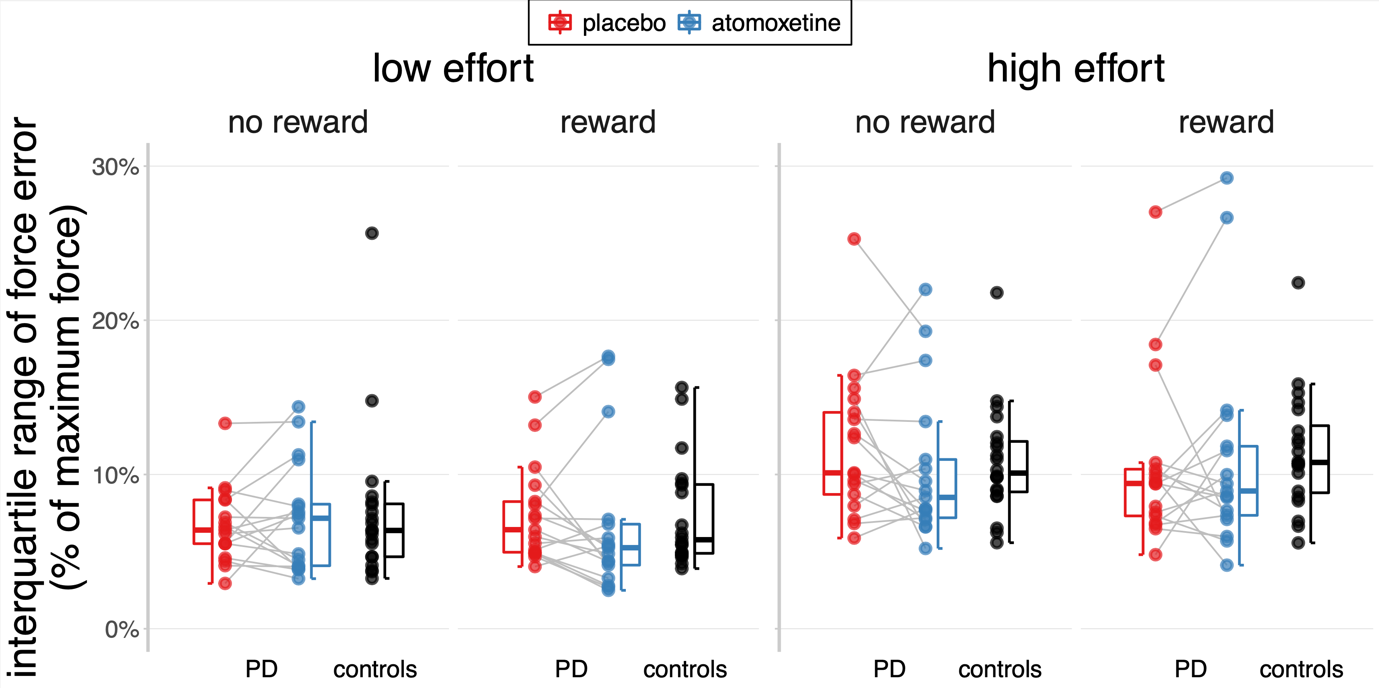


**Fig B | Task variability (interquartile range of force error) plotted as a function of effort, reward, group, and drug.** Dots represent individual participants, and boxplots represent the marginal distribution for a given condition. For the Parkinson’s disease group, the grey lines indicate within-subject change in the interquartile range of force error from placebo to atomoxetine. Box-plot elements: centre line, median; box limits, first and third quartiles; whiskers, most extreme observations within 1.5 × interquartile range from the box limits.


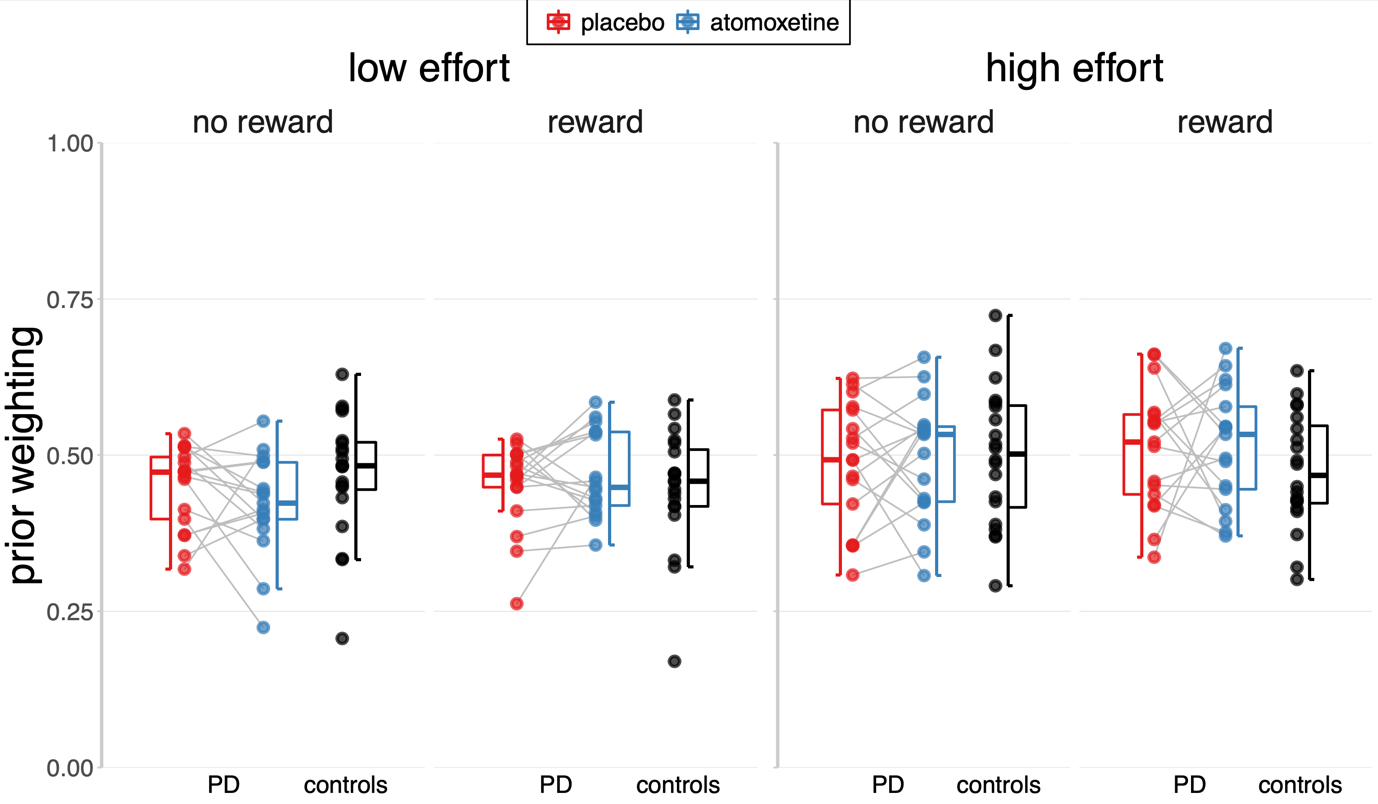


**Fig C | Prior weighting plotted as a function of effort, reward, group, and drug.** Dots represent individual participants, and boxplots represent the marginal distribution for a given condition. For the Parkinson’s disease group, the grey lines indicate within-subject change in prior weighting from placebo to atomoxetine. Box-plot elements: centre line, median; box limits, first and third quartiles; whiskers, most extreme observations within 1.5 × interquartile range from the box limits.

**Table A | Estimated marginal means of median force error by group, effort, and reward.**

|  |  |  | *M* | 95% CI |
| --- | --- | --- | --- | --- |
| PD (placebo) | Low Effort | No Reward | 1.18% | -0.62%, 2.99% |
|  |  | Reward | 1.01% | -0.80%, 2.81% |
|  | High Effort | No Reward | -3.42% | -5.22%, -1.61% |
|  |  | Reward | -2.81% | -4.61%, -1.00% |
| Controls | Low Effort | No Reward | 2.32% | 0.57%, 4.06% |
|  |  | Reward | 2.03% | 0.28%, 3.77% |
|  | High Effort | No Reward | -1.46% | -3.21%, 0.28% |
|  |  | Reward | -1.55% | -3.29%, 0.20% |

**Table B | Estimated marginal means of median force error by drug, effort, and reward.**

|  |  |  | *M* | 95% CI |
| --- | --- | --- | --- | --- |
| PD (placebo) | Low Effort | No Reward | 1.13% | -0.72%, 2.97% |
|  |  | Reward | 0.96% | -0.89%, 2.80% |
|  | High Effort | No Reward | -3.47% | -5.32%, -1.62% |
|  |  | Reward | -2.86% | -4.71%, -1.02% |
| PD (atomoxetine) | Low Effort | No Reward | 2.09% | 0.24%, 3.93% |
|  |  | Reward | 1.36% | -0.48%, 3.21% |
|  | High Effort | No Reward | -2.16% | -4.01%, -0.32% |
|  |  | Reward | -2.10% | -3.94%, -0.25% |

**Table C | Estimated marginal means of interquartile range of force error by group, effort, and reward.**

|  |  |  | *M* | 95% CI |
| --- | --- | --- | --- | --- |
| PD (placebo) | Low Effort | No Reward | 6.71% | 4.79%, 8.63% |
|  |  | Reward | 7.37% | 5.46%, 9.29% |
|  | High Effort | No Reward | 11.80% | 9.88%, 13.72% |
|  |  | Reward | 10.57% | 8.65%, 12.49% |
| Controls | Low Effort | No Reward | 7.54% | 5.67%, 9.41% |
|  |  | Reward | 7.36% | 5.49%, 9.23% |
|  | High Effort | No Reward | 10.83% | 8.97%, 12.70% |
|  |  | Reward | 11.45% | 9.58%, 13.32% |

**Table D | Estimated marginal means of interquartile range of force error by drug, effort, and reward.**

|  |  |  | *M* | 95% CI |
| --- | --- | --- | --- | --- |
| PD (placebo) | Low Effort | No Reward | 6.71% | 4.40%, 9.01% |
|  |  | Reward | 7.37% | 5.06%, 9.68% |
|  | High Effort | No Reward | 11.79% | 9.48%, 14.10% |
|  |  | Reward | 10.56% | 8.25%, 12.87% |
| PD (atomoxetine) | Low Effort | No Reward | 7.24% | 4.93%, 9.55% |
|  |  | Reward | 6.71% | 4.40%, 9.02% |
|  | High Effort | No Reward | 10.37% | 8.06%, 12.68% |
|  |  | Reward | 11.21% | 8.90%, 13.52% |

**Table E | Estimated marginal means of prior weighting by group, effort, and reward.**

|  |  |  | *M* | 95% CI |
| --- | --- | --- | --- | --- |
| PD (placebo) | Low Effort | No Reward | -0.32 | -0.79, 0.16 |
|  |  | Reward | -0.25 | -0.72, 0.22 |
|  | High Effort | No Reward | 0.13 | -0.34, 0.60 |
|  |  | Reward | 0.41 | -0.06, 0.88 |
| Controls | Low Effort | No Reward | 6.73 × 10^-4^ | -0.44, 0.44 |
|  |  | Reward | -0.27 | -0.71, 0.17 |
|  | High Effort | No Reward | 0.29 | -0.15, 0.73 |
|  |  | Reward | 4.82 × 10^-3^ | -0.43, 0.44 |

*Note*: The ANOVA model from which the marginal means and confidence intervals were estimated included performance variability as a between-subjects covariate of no interest.

**Table F | Estimated marginal means of prior weighting by drug, effort, and reward.**

|  |  |  | *M* | 95% CI |
| --- | --- | --- | --- | --- |
| PD (placebo) | Low Effort | No Reward | -0.29 | -0.74, 0.15 |
|  |  | Reward | -0.26 | -0.70, 0.18 |
|  | High Effort | No Reward | 0.15 | -0.30, 0.59 |
|  |  | Reward | 0.41 | -0.04, 0.85 |
| PD (atomoxetine) | Low Effort | No Reward | -0.60 | -1.04, -0.16 |
|  |  | Reward | -0.07 | -0.51, 0.38 |
|  | High Effort | No Reward | 0.23 | -0.21, 0.67 |
|  |  | Reward | 0.44 | -2.46 × 10^-3^, 0.89 |

*Note*: The ANOVA model from which the marginal means and confidence intervals were estimated included performance variability as a between-subjects covariate of no interest.
