# Supplementary material for: Noradrenergic deficits contribute to apathy in Parkinson’s disease through the precision of expected outcomes
Source: PLoS Comput Biol. 2022 May 9;18(5):e1010079. doi: 10.1371/journal.pcbi.1010079 (PMC9119485; doi:10.1371/journal.pcbi.1010079)
Supplement: S5 Text — Fig A. Parameter recovery of prior weighting. (A) Data-generating prior weighting plotted against the estimated (i.e., recovered) prior weighting. The diagonal (identity) line represents perfect parameter recovery. For a given data-generating prior weighting value, the dot represents the median of the prior weighting estimates across 2000 simulations of estimation errors; the vertical error bars represent the 95% quantile intervals of the prior weighting estimates. The inset histogram illustrates the distribution of the difference between the median estimated prior weighting and data-generating prior weighting. (B, C) Examples of data simulations for a relatively low data-generating value of prior weighting (B; orange dot and error bar in A) and a relatively high data-generating value of prior weighting (C; green dot in A). (DOCX) [file pcbi.1010079.s010.docx]

**S5 Text: Parameter recovery of prior weighting**

We performed a parameter recovery analysis to examine the identifiability of prior weighting, as estimated by a linear mixed effects model of estimation error against performance error. To this end, we first simulated 100 datasets of performance errors, closely following the design of the visuomotor task. Each simulated dataset consisted of four blocks of 30 trials, with one block for each condition of the effort and reward manipulations. For each simulated dataset $i$ and condition $k$, the vector of 30 simulated performance errors $\boldsymbol{x}_{\mathrm{sim}}^{(i,k)}$ was drawn from a truncated normal distribution:

$$\begin{aligned} \boldsymbol{x}_{\mathrm{sim}}^{\left( i, k \right)}\sim\mathcal{N}_{\left( x_{\min},x_{\max} \right)}\left( \mu^{(k)}, \sigma^{(k)} \right)\#\left( 1 \right) \end{aligned}$$

where $\mu^{(k)}$ and $\sigma^{(k)}$ represent the observed group-level mean and standard deviation of performance error for condition $k$, and the truncation points $x_{\min}$ and $x_{\max}$represent the bounds on performance error, given the target position for condition $k$ and the limits of the laptop screen.

We then assigned the standard deviations of the prior and sensory evidence distributions, $\sigma_{\mathrm{prior}}^{(i)}$ and $\sigma_{\mathrm{evidence}}^{(i)}$, which jointly constitute the prior weighting term $w_{\mathrm{prior}}^{(i)}$ and the posterior standard deviation $\sigma_{\hat{x}}^{(i)}$. Given that the standard deviations of the prior and sensory evidence are strongly correlated with the variance of performance errors [1,2], they were drawn from truncated normal distributions whose mean and standard deviation depended on the standard deviation of the simulated performance errors, $\sigma_{\mathrm{sim}}^{(i)}$:

$$\sigma_{\mathrm{prior}}^{\left( i \right)}\sim\mathcal{N}_{+}\left( \sigma_{\mathrm{sim}}^{\left( i \right)}, {0.5\cdot\sigma}_{\mathrm{sim}}^{\left( i \right)} \right)$$

$$\sigma_{\mathrm{evidence}}^{\left( i \right)}\sim\mathcal{N}_{\left( 0.5\cdot\sigma_{\mathrm{sim}}^{\left( i \right)},1.5\cdot\sigma_{\mathrm{sim}}^{\left( i \right)} \right)}\left( \sigma_{\mathrm{sim}}^{\left( i \right)},0.3\cdot\sigma_{\mathrm{sim}}^{\left( i \right)} \right)$$

$$\begin{aligned} w_{\mathrm{prior}}^{\left( i \right)}=\frac{\left[ \sigma_{\mathrm{evidence}}^{\left( i \right)} \right]^{2}}{\left[ \sigma_{\mathrm{evidence}}^{\left( i \right)} \right]^{2}+\left[ \sigma_{\mathrm{prior}}^{\left( i \right)} \right]^{2}} \\ \sigma_{\hat{x}}^{(i)}=\frac{\left[ \sigma_{\mathrm{evidence}}^{\left( i \right)} \right]^{2}\cdot\left[ \sigma_{\mathrm{prior}}^{\left( i \right)} \right]^{2}}{\left[ \sigma_{\mathrm{evidence}}^{\left( i \right)} \right]^{2}+\left[ \sigma_{\mathrm{prior}}^{\left( i \right)} \right]^{2}} \#(2) \end{aligned}$$

Note that we constrained the standard deviation of sensory evidence to be within a relatively close range of the standard deviation of the simulated performance errors, $\left[ 0.5\cdot\sigma_{\mathrm{sim}}^{\left( i \right)},1.5\cdot\sigma_{\mathrm{sim}}^{\left( i \right)} \right]$, since we expected individual differences in prior weighting to be primarily driven by variation in the standard deviation of the prior [1].

For each simulated dataset $i$, we pseudorandomly selected 10 trials within each block $k$ as estimation trials, thus yielding 40 estimation trials. For a given estimation trial $n$, the posterior estimate of the final ball position is a precision-weighted combination of the prior (centred on the target position) and the likelihood (centred on the simulated true final ball position):

$$\begin{aligned} \hat{x}^{(i,n)}=w_{\mathrm{prior}}^{\left( i \right)}\cdot x_{\mathrm{target}}^{\left( i,k \right)}+\left( 1-w_{\mathrm{prior}}^{\left( i \right)} \right)\cdot x_{\mathrm{ball}}^{\left( i,n \right)} \#(3) \end{aligned}$$

Considering the posterior standard deviation of this optimal estimate, $\sigma_{\hat{x}}^{(i)}$, we simulated the estimated final ball position by drawing from the trial-wise posterior distribution:

$$\begin{aligned} x_{\mathrm{estimate}}^{\left( i,n \right)}\mathcal{\sim N}\left( \hat{x}^{\left( i,n \right)},\sigma_{\hat{x}}^{\left( i \right)} \right) \#\left( 4 \right) \end{aligned}$$

In line with our task design, we pseudorandomly determined the position of an estimation grid, consisting of a set of 12 evenly spaced response options $R$, for each estimation trial $n$. The simulated response $y$ for estimation trial $n$ is the response option $r$ that minimises the absolute distance from the simulated estimate of the final ball position:

$$\begin{aligned} y^{\left( i,n \right)}=\underset{r\in R}{\mathrm{argmin}} \left| r-x_{\mathrm{estimate}}^{\left( i,n \right)} \right| \#\left( 5 \right) \end{aligned}$$

The estimation errors are then given as the difference between the simulated response and the response option centred on the true final ball position. The z-scored estimation errors were regressed against the z-scored performance errors, and the negative of the regression coefficient was taken as the estimate of prior weighting. To examine the uncertainty of parameter recovery, we repeated the simulation of the estimated final ball position (Equation 4) 2000 times. Thus, for each data-generating setting of prior weighting $w_{\mathrm{prior}}^{(i)}$ and the corresponding vector of $N$ simulated estimation trials, we obtained a $2000\times N$ matrix of plausible estimates of the final ball positions. We performed the regression analysis of estimation error against performance error separately for each of the 2000 repetitions, yielding a distribution of plausible estimates of prior weighting for each of the 100 data-generating prior weighting values.

Parameter recovery performance is illustrated below in Fig A. The data-generating and median estimated prior-weighting values were very strongly correlated (*r*_(98)_ = 0.94, *t* = 27.37, *p* < .001; *BF* = 3.54 × 10^42^), indicating good parameter recovery. There was a small positive bias, such that the estimates of prior weighting tended to be slightly higher than the data-generating values (mean bias = 0.04, SD = 0.08).


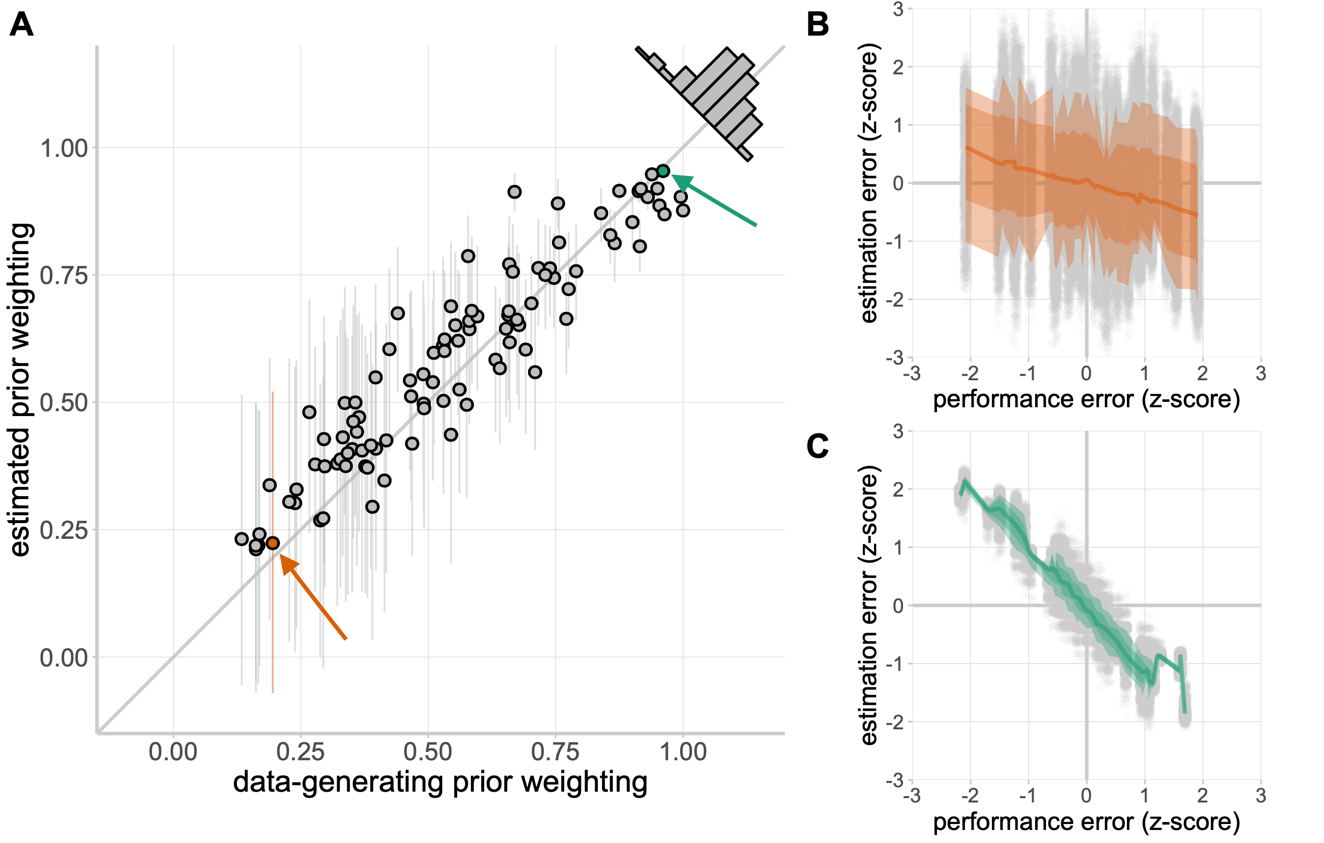


**Fig A | Parameter recovery of prior weighting.** (A) Data-generating prior weighting plotted against the estimated (i.e., recovered) prior weighting. The diagonal (identity) line represents perfect parameter recovery. For a given data-generating prior weighting value, the dot represents the median of the prior weighting estimates across 2000 simulations of estimation errors; the vertical error bars represent the 95% quantile intervals of the prior weighting estimates. The inset histogram illustrates the distribution of the difference between the median estimated prior weighting and data-generating prior weighting. (B, C) Examples of data simulations for a relatively low data-generating value of prior weighting (B; orange dot and error bar in A) and a relatively high data-generating value of prior weighting (C; green dot in A).

**References**

1. Hezemans FH, Wolpe N, Rowe JB. Apathy is associated with reduced precision of prior beliefs about action outcomes. J Exp Psychol Gen. 2020 [cited 17 May 2020]. doi:10.1037/xge0000739

2. Wolpe N, Wolpert DM, Rowe JB. Seeing what you want to see: priors for one’s own actions represent exaggerated expectations of success. Front Behav Neurosci. 2014;8. doi:10.3389/fnbeh.2014.00232
