# Supplementary material for: Noradrenergic deficits contribute to apathy in Parkinson’s disease through the precision of expected outcomes
Source: PLoS Comput Biol. 2022 May 9;18(5):e1010079. doi: 10.1371/journal.pcbi.1010079 (PMC9119485; doi:10.1371/journal.pcbi.1010079)
Supplement: S6 Text — (DOCX) [file pcbi.1010079.s011.docx]

**S6 Text: Details of statistical software**

The statistical analyses were implemented in R version 3.6.1 [1] using the ‘tidyverse’ version 1.3.0 [2] and ‘tidybayes’ version 2.3.1 [3] packages for data organisation and visualisation; the ‘lme4’ version 1.1-21 [4], ‘robustlmm’ version 2.4-2 [5], and ‘afex’ version 0.25-1 [6] packages for fitting linear mixed effects models and ANOVA; the ‘emmeans’ version 1.5.3 [7] and ‘interactions’ version 1.1.3 [8] packages for post-hoc analyses; and the ‘BayesFactor’ version 0.9.12-4.2 [9] and ‘bayestestR’ version 0.6.0 [10] packages for Bayes Factor analyses. The hierarchical Bayesian modelling was implemented in Stan [11] using the ‘rstan’ version 2.21.2 [12] interface package. Model comparison was performed with the ‘loo’ version 2.3.1 package [13].

**References**

1. R Core Team. R: A Language and Environment for Statistical Computing. Vienna, Austria: R Foundation for Statistical Computing; 2019. Available: https://www.R-project.org/

2. Wickham H, Averick M, Bryan J, Chang W, McGowan L, François R, et al. Welcome to the Tidyverse. J Open Source Softw. 2019;4: 1686. doi:10.21105/joss.01686

3. Kay M. tidybayes: Tidy Data and Geoms for Bayesian Models. Zenodo; 2020. Available: https://zenodo.org/record/3740308#.XutG2WozbEY

4. Bates D, Mächler M, Bolker B, Walker S. Fitting Linear Mixed-Effects Models Using **lme4**. J Stat Softw. 2015;67. doi:10.18637/jss.v067.i01

5. Koller M. robustlmm: An R Package for Robust Estimation of Linear Mixed-Effects Models. J Stat Softw. 2016;75. doi:10.18637/jss.v075.i06

6. Singmann H, Bolker B, Westfall J, Aust F, Ben-Shachar MS. afex: Analysis of Factorial Experiments. 2020. Available: https://CRAN.R-project.org/package=afex

7. Lenth R, Singmann H, Love J, Buerkner P, Herve M. emmeans: Estimated Marginal Means, aka Least-Squares Means. 2020. Available: https://CRAN.R-project.org/package=emmeans

8. Long JA. interactions: Comprehensive, User-Friendly Toolkit for Probing Interactions. 2019. Available: https://cran.r-project.org/package=interactions

9. Morey RD, Rouder JN. BayesFactor: Computation of Bayes Factors for Common Designs. 2018. Available: https://CRAN.R-project.org/package=BayesFactor

10. Makowski D, Ben-Shachar M, Lüdecke D. bayestestR: Describing Effects and their Uncertainty, Existence and Significance within the Bayesian Framework. J Open Source Softw. 2019;4: 1541. doi:10.21105/joss.01541

11. Carpenter B, Gelman A, Hoffman MD, Lee D, Goodrich B, Betancourt M, et al. Stan: A Probabilistic Programming Language. J Stat Softw. 2017;76. doi:10.18637/jss.v076.i01

12. Stan Development Team. RStan: the R interface to Stan. 2019. Available: http://mc-stan.org/

13. Vehtari A, Gelman A, Gabry J. Practical Bayesian model evaluation using leave-one-out cross-validation and WAIC. Stat Comput. 2017;27: 1413–1432. doi:10.1007/s11222-016-9696-4
