# Supplementary material for: Noradrenergic deficits contribute to apathy in Parkinson’s disease through the precision of expected outcomes
Source: PLoS Comput Biol. 2022 May 9;18(5):e1010079. doi: 10.1371/journal.pcbi.1010079 (PMC9119485; doi:10.1371/journal.pcbi.1010079)
Supplement: Attachment — Submitted filename: Hezemans_PLOS_CB_responseletter.docx [file pcbi.1010079.s012.docx]

**Reviewer 1**

**Reviewer Comment 1.1**

This is an interesting study in which the authors use a clever task that allows them to distinguish subjects' precision of their prior expectations (about a target's movement) from the precision of the sensory likelihood (from the applied force to the target). They collect data from n=17 Parkinson's patients and controls; the former are also given Atomoxetine and placebo in a within-subject manipulation, and 7T imaging of their Locus Coereleus. The authors found a negative correlation between prior/likelihood weighting and apathy, and an interaction such that subjects with lower LC integrity showed a positive effect of ATO on this weighting (higher LC integrity resulted in a negative effect). They are careful to control for other factors, which do not affect the results. Overall the topic is interesting and clinically important, and the methods are rigorous.

**Author Reply 1.1**

We are grateful for the Reviewer’s positive remarks about our work. We appreciate their constructive feedback, which has encouraged us to refine our interpretations, with several changes in the Discussion and Materials and Methods sections, as set out below.

**Reviewer Comment 1.2**

There are some weaknesses: the main ones being that the sample size is fairly small (n=17), controls did not receive ATO or MRI scanning, Atomoxetine also has effects on DA as well as NA, and the PD patients were not any more apathetic than controls. This makes it difficult to draw firm conclusions about mechanisms of apathy in PD specifically. But the relationship of ATO effects on prior precision to LC integrity is certainly of interest.

**Author Reply 1.2**

Several issues are raised here. We appreciate the Reviewer’s excitement about our reported relationship between the effect of atomoxetine on prior precision and locus coeruleus integrity. We address the Reviewer’s important concerns separately below.

**Reviewer Comment 1.2.1**

The sample size is fairly small (N=17).

**Author Reply 1.2.1**

A larger sample size can often be helpful. However, our study focused on hypothesis-based analyses of within-subject effects of atomoxetine on prior weighting, for which we expected relatively large effect sizes given our prior work (e.g. Ye et al., 2015; O’Callaghan et al., 2021). We report estimates of effect sizes (standardised regression coefficients) and Bayes factors as continuous metrics of statistical evidence, in line with frequently reported best practice guidelines (e.g., Cumming, 2014). The use of Bayesian statistics is especially relevant as the frequentist type II error does not arise. Rather, the Bayes factor above 3 or below 1/3 is indicative that the data have sufficient precision to draw inferences. Insufficient “power” (in the lay sense) is likely to lead to indeterminate Bayes factors (1/3 ≤ BF ≤ 3), indicative of the lack of evidence for either the null or alternative hypothesis. Moreover, to facilitate conceptual replications and extensions of our work, we have made our code and data freely available through the Open Science Framework (<https://osf.io/mry4f/?view_only=9201a50a0b094c45bbe8cacac44a5c41>).

In the Discussion section of the revised manuscript, we have revised our acknowledgement of the limited sample size as follows (changes highlighted in yellow):

“This study has limitations. First, although we collected a comprehensive dataset for each individual participant, incorporating various demographic, behavioural, and neuropharmacological measures, the total number of participants is modest. Our study design was therefore not well-suited for data-driven analyses [91] or predictive modelling [92], and may have been prone to type II error for frequentist statistical inferences on small effects. We focused on the analyses of theory-driven models, with reporting of effect sizes and complementary Bayesian statistics.”

**Reviewer Comment 1.2.2**
Controls did not receive ATO.

**Author Reply 1.2.2**

Control participants were tested in a single session of MRI scanning and behavioural experiments, and they did not undergo the drug manipulation. The purpose of the control group was to provide benchmark normative data for the novel task and MRI of locus coeruleus. This study design is in line with previous published work by our lab and by others (e.g. Kehagia et al., 2014; Ye et al., 2015; Rae et al., 2016).

In general, clinical trials have a placebo intervention (as we have) but not a group without the relevant disease or condition. We designed the current study to test a mechanistic hypothesis about noradrenergic contributions to apathy in the context of Parkinson’s disease. Administering atomoxetine to healthy controls would not have helped us to answer the research questions. For our hypotheses related to the impact of LC degeneration in Parkinson’s disease, the lack of a drug manipulation for the control group is not a *weakness* of our study design *per se*. To study the effects of atomoxetine on prior weighting in healthy participants would be a fundamentally different research project. While such a project might be of interest, it would not be relevant to the study of restorative effects of drug in the context of disease.

**Reviewer Comment 1.2.3**
Controls did not receive MRI scanning.

**Author Reply 1.2.3**

On the contrary, the control group did undergo the same ultra-high field MRI scanning as the Parkinson’s disease group (as noted in the Materials and Methods, lines 529 – 531).

We have separately reported between-group comparisons of locus coeruleus integrity (O’Callaghan et al., 2021), using data from a largely overlapping sample of participants. The current study focused on individual differences in the effect of noradrenaline/atomoxetine on prior weighting. As such, between-group comparisons of locus coeruleus integrity were not of primary interest, and we therefore cite O’Callaghan et al. (2021) rather than re-report the between-group MRI results in the current manuscript.

We now clarify this in the Materials and Methods section, in the subsection Image Processing and Locus Coeruleus Integrity, to explicitly refer the reader to O’Callaghan et al. (2021) for further details regarding the locus coeruleus imaging:

“To estimate the locus coeruleus integrity, we applied the independent locus coeruleus atlas to each participant’s CNR map, and then calculated the mean CNR value across the whole structure. (…) Comparisons of locus coeruleus CNR between the Parkinson’s disease and control groups are reported in detail in O’Callaghan et al. [62].”

**Reviewer Comment 1.2.4**
Atomoxetine has effects on DA as well as NA.

**Author Reply 1.2.4**

We agree with the Reviewer that it can be challenging to disentangle the noradrenergic *versus* dopaminergic effects of atomoxetine. Due to the limited expression of dopamine transporters in the prefrontal cortex (Sesack et al., 1998), a portion of dopamine reuptake is mediated by the noradrenaline transporter (Wayment et al., 2001; Seamans et al., 2004). Thus, by blocking the noradrenaline transporter, atomoxetine could increase both noradrenaline and dopamine levels in the prefrontal cortex (Bymaster et al., 2002; Devoto & Flore, 2006). For these reasons, pharmacological manipulations like atomoxetine can affect both the noradrenergic and dopaminergic systems to varying degrees. The question is then a matter of degree and likelihood. For example, in contrast to methylphenidate, atomoxetine does not increase extracellular dopamine in the striatum or posterior cortex (Bymaster et al., 2002; Swanson et al., 2006). In the prefrontal cortex, 1-3mg/kg atomoxetine causes a ~3-fold increase in extracellular noradrenaline, but at 0.3mg/kg in rats, the prefrontal cortical increase in dopamine was smaller, just 50%. While dose equivalence across species is challenging (see e.g. FDA guidelines summarised in Nair et al., 2016), the oral 40mg in our human participants is unlikely to be equivalent to the >1mg/kg required in rats for substantial dopamine increases. Even at 3mg/kg, the effect of atomoxetine on noradrenaline in rat prefrontal cortex is nearly twice that of dopamine (Umehara et al., 2013). In human studies of other goal-directed behaviours, such as the stop-signal task, drugs that act on both noradrenergic and dopaminergic systems affect behaviour, whereas selective dopaminergic drugs do not (discussed in O’Callaghan et al., 2021). The balance of probability is therefore for atomoxetine’s effect to be exerted in this study via predominantly noradrenergic rather than dopaminergic systems.

We appreciate the opportunity to highlight this important issue, and have therefore revised the relevant section in the Discussion as follows:

“This study has limitations. (…) Third, we acknowledge the psychopharmacological complexity of medicated Parkinson’s disease patients and atomoxetine. Due to the limited expression of dopamine transporters in the prefrontal cortex [94], a portion of dopamine reuptake is mediated by the noradrenaline transporter [95,96]. Thus, by blocking the noradrenaline transporter, atomoxetine can increase both noradrenaline and dopamine levels in the prefrontal cortex [89,97]. It can be challenging to disentangle the noradrenergic versus dopaminergic effects of atomoxetine. Noradrenaline and dopamine are closely related neuromodulators that are synthesised from a shared metabolic pathway. They can be co-released from the same noradrenergic terminals in the prefrontal cortex [98,99]. Furthermore, locus coeruleus activity can alter midbrain dopamine cell firing [100] and contribute to dopamine release in the hippocampus [101]. Thus, we cannot wholly exclude the possibility that atomoxetine-induced changes in prior weighting partially reflected dopaminergic effects. (…) Future studies could opt for a crossover design with both noradrenergic and dopaminergic agents, to enhance the specificity of findings.”

**Reviewer Comment 1.2.5**
The PD patients were not any more apathetic than controls. This makes it difficult to draw firm conclusions about mechanisms of apathy in PD specifically.

**Author Reply 1.2.5**

We acknowledge that trait apathy, as measured with the Starkstein Apathy Scale, was on average not significantly more severe among participants with Parkinson’s disease compared to controls (as noted in Table 1 and the S1 Text). However, our statistical inferences do not rely on the Parkinson’s disease group being severely apathetic, since we modelled the relationship between apathy and prior weighting as continuous variables. In other words, our analysis approach focused on individual differences in apathy in relation to prior weighting, as opposed to comparisons of apathetic *versus* non-apathetic participants. Nevertheless, we accept that it remains to be proven whether our findings generalise to more severe apathy in other patients with Parkinson’s disease.

We have now incorporated this point into the limitations paragraph of the revised Discussion as follows:

“This study has limitations. (…) Second, the participants with Parkinson’s disease were not severely apathetic, as the group mean score on the Apathy Scale fell below conventional cut-off scores for clinically significant apathy [1,59], and there was no statistically significant difference in apathy between the Parkinson’s disease and control groups. We adopted a dimensional approach [17,93], focusing on individual differences rather than group contrasts in the mechanisms underlying apathy. Nevertheless, we recognise that it remains to be proven whether the current findings generalise to more severely apathetic cohorts.”

**Reviewer Comment 1.3**

p15 - The authors are careful to say that these findings "suggest" a contributory mechanism to apathy in PD, but I think they should also explicitly stress that these PD patients were not apathetic - their apathy scores were no different to controls'. So this makes it difficult to extrapolate the findings to pathological levels of apathy. I would therefore rephrase the subsequent conclusion "this study provides evidence for a noradrenergic role in apathy in Parkinson’s disease".

**Author Reply 1.3**

As discussed in Author Reply 1.2.5, we acknowledge that the Parkinson’s disease group in our study was not severely apathetic, and we have revised our Discussion to emphasise this point. However, people with mild apathy in the context of Parkinson’s are of clinical interest. These patients are likely to have a poorer prognosis, with reduced quality of life and greater caregiver burden. This group therefore represents an important cohort who could potentially benefit from atomoxetine therapy. Furthermore, other groups have reported similar results were PD patients may be impaired on experimental measures of effort/motivation, even in the absence of a clinical syndrome of apathy (e.g. Chong et al., 2015). We believe that is more constructive to approach apathy as a dimensional rather than categorical construct. From this perspective, our findings support the idea that an apathy-dependent cognitive mechanism (i.e., prior weighting) was modulated by noradrenaline in a baseline-dependent manner. At the same time, we recognise and appreciate the Reviewer’s call for nuance in our interpretations, and have therefore rephrased the conclusion as follows:

“In conclusion, this study provides preliminary evidence for a noradrenergic role in apathy in Parkinson’s disease, via the precision weighting of prior beliefs about action outcomes.”

**Reviewer Comment 1.4**

p3/16 - There seems to be a tension between some of the evidence about noradrenaline (NA). At the bottom of p3, lots of evidence is cited that NA relates to (phasic) unexpected uncertainty or (tonic) volatility in the environment, i.e. evidence that the model must be updated - hence priors should be weakened, in both cases. But how does this square with the finding that ATO increases the precision of priors, in those with a less intact LC? There is some rather intricate speculation in the Discussion about phasic/tonic LC firing ratios. But to me this indicates that the subjects with *intact* LC show the most intuitive result: that ATO *reduces* prior precision (as NA ought to if it encodes unexpected uncertainty). This makes me wonder whether the opposite ATO effects in those with less intact LC are in fact dopaminergic effects, unmasked by the impaired LC system? This makes more sense to me than positing an inverted-U or altered phasic/tonic relationships. Impossible to tell from this work of course, but I think this possibility should be mentioned a bit more clearly in line 430 or earlier.

**Author Reply 1.4**

The computational role of the locus coeruleus noradrenaline system is a topic of ongoing debate, and we appreciate the Reviewer’s thought-provoking interpretation of our findings. Existing theoretical frameworks, relying primarily on non-human animal studies and computational modelling, have likened noradrenaline activity to neural gain (e.g., Usher et al., 1999; Aston-Jones & Cohen, 2005), or surprise about state transitions (Dayan & Yu, 2006; Sales et al., 2019). However, considering psychopharmacological and pupillometry work with human participants, the directionality of such relationships is not straightforward, especially when one considers baseline-dependency of neuromodulators (Cools, 2019; Aston-Jones & Cohen, 2005; Jepma et al., 2016).

Several studies provide evidence for greater reliance on sensory evidence following an increase of noradrenaline (e.g. Nassar et al., 2012; Lawson et al., 2021), but other studies suggest the opposite effect. For example, Eldar and colleagues (2013) found that greater neural gain (as indexed through pupillometry) was associated with a greater influence of “individual *priors* [emphasis added] or attentional predispositions (so-called learning styles) on trial-and-error learning behavior”. Furthermore, Hauser et al. (2017) found that a noradrenaline blockade by propranolol caused metacognitive estimates of performance in a perceptual decision-making task to be more in line with true performance. Put differently, a *reduction* of noradrenaline led to *greater* reliance on sensory evidence for one’s own performance.

We have framed our interpretation of the relationship between locus coeruleus integrity and the effect of atomoxetine on prior weighting around the hypothesis of an inverted-U pattern of noradrenaline responses. This hypothesis has strong support in previous work, and we believe it is the most parsimonious account of otherwise opposing effects of atomoxetine on cognition and behaviour (Aston-Jones & Cohen, 2005; Robbins, 2000). For people with a more impaired locus coeruleus (and consequent noradrenergic depletion), atomoxetine pushed them towards a more optimal range of function, putatively by increasing forebrain noradrenaline and the phasic-to-tonic ratio of locus coeruleus firing (Bari & Aston-Jones, 2013). In contrast, for people with a more intact locus coeruleus, that same dose of atomoxetine pushed them outside of an optimal range with excessive tonic noradrenaline release. We speculate that for people with reduced locus coeruleus integrity, the atomoxetine-induced increase in phasic locus coeruleus activity increased the reliance on prior beliefs for inferences about task performance (c.f. Eldar et al., 2013; Hauser et al., 2017).

We agree that the possibility of dopaminergic contributions to the effects of atomoxetine cannot be fully excluded. Please see Author Reply 1.2.4 above.

**Reviewer Comment 1.5**

p22 - Can the authors explain a bit more why the 2x2 effort/reward manipulation was included if it was not being used? They just state "we did not analyse these factors further, following Hezemans et al. [43]" but this is a bit thin. E.g. why not check for an effect of Atomoxetine on these factors? Is there insufficient power with n=17 subjects?

**Author Reply 1.5**

We thank the Reviewer for raising this issue. When designing the study, we aimed to replicate the visuomotor task from our previous study in healthy young controls (Hezemans et al., 2020), with the exception of a reduced number of trials to accommodate an older-age clinical cohort. The results from this previous study suggested a strong relationship between trait apathy and prior weighting. However, within participants, the effects of effort and reward on prior weighting were relatively small, and not significantly associated with apathy (see the Discussion in Hezemans et al., 2020, for further treatment of these results).

For the current study, our hypotheses focused on participant-level estimates of prior weighting, and their modulation by noradrenaline. However, we recognise the potential interest in the effects of the effort and reward manipulations. For full reporting of all experimental manipulations, the revision of the manuscript now includes an additional supplementary file that describes the effects of effort and reward on indices of basic task performance as well as prior weighting. In the revised manuscript, we refer the reader to this supplementary file as follows:

“For consistency with the original study design in Hezemans et al. [43], the task additionally featured experimental manipulations of effort and reward. (…) Following the results of Hezemans et al. [43], showing minimal effects of effort and reward on prior weighting that were not associated with apathy, we report the effects of these conditions on behaviour in the S4 text.”

**Reviewer Comment 1.6**

Minor comment: p4 - I would frame the opposite of 'active' inference as perceptual inference, not 'passive' inference.

**Author Reply 1.6**

We have rephrased the relevant paragraph as follows:

“With predictive processing, the brain optimises a probabilistic model of its environment, minimising ‘surprise’ or prediction error via action and perception [44,45]. The balance between active and passive (sensory) inference depends on the relative precision of prior beliefs and sensory evidence. (…) If precision on the priors is not sufficiently high, passive (sensory) inference occurs by adjusting perceptual priors, and no goal-directed action is necessary.”

**Reviewer 2**

**Reviewer Comment 2.1**

I am grateful for the opportunity to review this excellent paper. I very much enjoyed reading it, and think this is a great example of computational neurology. It is difficult to find much to improve on, but there are a few points that would benefit from clarification.

**Author Reply 2.1**

We thank the Reviewer for their kind remarks. We are delighted with their excitement about our manuscript.

**Reviewer Comment 2.2**

I thought the method for estimating the prior weighting based upon the slope between the two forms of error was very convincing in its simplicity. However, it would be useful to clarify whether this slope was calculated only for those trials in which the trajectory was not shown. I will assume this is the case in what follows. The reason this is important is that the sensory evidence is different between the conditions where the trajectory is and is not shown, and the performance error from those when it is cannot really be inserted into Equation 3, which computes the estimation error on the assumption of no visual trajectory data (the estimation will clear be different when visual data are available).

**Author Reply 2.2**

We appreciate the Reviewer’s positive words about our modelling approach. We confirm that the slope of performance error on estimation error (i.e., the negative of prior weighting) was only estimated for trials where the ball trajectory was not shown to the participant.

We have emphasised this point in the revised Results section as follows:

“We used a linear mixed effects model to estimate the relationship between estimation error and performance error, allowing the slope (i.e., negative prior weighting) to vary for each testing session for each participant. Note that this model was only fit to the subset of trials where the ball’s visual trajectory was not shown (i.e., estimation trials, see Materials and Methods for details). Thus, participants could not have used visual information to infer the ball’s final position.”

**Reviewer Comment 2.3**

Following from the above, could the author's comment on the sensory modalities assumed to contribute to the evidence? If not visual, I presume this will largely be tactile and proprioceptive data associated with the finger press. If so, this might be something the authors could make more of. As their focus is on a neurological condition often characterised as a movement disorder, it seems a very good experimental design that focuses on sensory modalities that result from self-generated movements.

**Author Reply 2.3**

There may be diverse sources of trial-wise sensory evidence in our visuomotor experiment. These are likely to include ascending tactile and proprioceptive input associated with the finger press, and we appreciate the Reviewer’s encouragement to expand on this idea. At the same time, our modelling approach deliberately focused on general principles of perceptual inference in the context of effortful, goal-directed behaviour, without commitment to a specific modality or algorithmic implementation in the brain. We referred to the prior belief and sensory evidence as metaphors for an undoubtedly complex, hierarchical network of representations at multiple levels of abstraction. Therefore, we refrain from making specific claims about the information content of sensory evidence in our visuomotor task. The Reviewer may want to cross-reference with Reviewer 3, who raised a number of points about the nature of sensory evidence and force production (Comment 3.2.1). We have added the following paragraph to the revised Discussion:

We used Bayesian inference as a principled framework to model the computational goal of perceptual inference in the context of effortful, goal-directed behaviour. However, we cannot comment directly on the exact algorithmic underpinnings of Bayesian perceptual inference in our experiment [105]. Following hierarchical models of predictive coding, one could decompose behaviour in our experiment into predictions at multiple levels of abstraction, from low-level proprioceptive predictions for movement to high-level multi-modal, domain-general beliefs [46,106-111]. Future work is needed to address how such a framework can accommodate the various stages of information processing that are thought to be involved in effort- and reward-based decision-making [112-114], including the weighting of action policies [13,15,53], initiating and sustaining an action [115], and evaluating and learning from the action outcomes.

**Reviewer Comment 2.4**

One difficulty is that the premise of this study is based upon apathy in Parkinson's disease, but it seems that the Parkinson's patients were no more apathetic than the controls. However, the authors highlight the association between locus coeruleus degeneration and apathy, and the interaction between prior weighting and the effect of pharmacological manipulations. Do the authors feel that this experiment could be practically adapted as a clinical test to identify potential responders to atomoxetine from a clinically heterogenous group?

**Author Reply 2.4**

Firstly, we acknowledge that our participants with Parkinson’s disease were not significantly more apathetic than our controls. However, we do not necessarily view this as a weakness of our study, as we approach the problem from a dimensional perspective (i.e., *relatively* more apathetic participants tended to have reduced prior weighting) as opposed to a categorical one (i.e., comparing “apathetic” *versus* “non-apathetic” participants). Nevertheless, we accept that it remains to be proven whether our findings generalise to those with more severe apathy in Parkinson’s disease, and have noted this point in our revised Discussion. The Reviewer may also want to cross-reference with Reviewer 1, who raised a similar point (Comments 1.2.5 and 1.3).

“This study has limitations. (…) Second, the participants with Parkinson’s disease were not severely apathetic, as the group mean score on the Apathy Scale fell below conventional cut-off scores for clinically significant apathy [1,59], and there was no overall difference in apathy between the Parkinson’s disease and control groups. We adopted a dimensional approach [17,93], focusing on individual differences rather than group contrasts in the mechanisms underlying apathy. Nevertheless, we recognise that it remains to be proven whether the current findings generalise to more severely apathetic cohorts.”

Secondly, we view the current study as a proof of concept that informs future clinical trials on the noradrenergic regulation of apathy. However, we would emphasise that locus coeruleus imaging was necessary to explain the mechanism of benefit of atomoxetine.

To that end, we have previously argued that locus coeruleus integrity could be used to stratify patients in future clinical trials of atomoxetine (O’Callaghan et al., 2021). Indeed, 7T imaging of the locus coeruleus is included in an ongoing clinical trial of atomoxetine in people with progressive supranuclear palsy for this purpose, in a planned exploratory analysis (“NORAPS”). Our behavioural experiment alone was not sufficient to identify potential responders to atomoxetine from a clinically heterogenous group. We designed our visuomotor task to target a latent cognitive parameter that we propose to be relevant to the mechanisms underlying apathy. Given prior theoretical work, we predicted that this cognitive parameter was modulated by noradrenaline in a baseline-dependent manner, and we suggest that this prediction is borne out by our data. We do not claim that performance in our visuomotor task is in itself a clinically useful measure of apathy (i.e., to detect instances of apathy in everyday behaviour). To determine the clinical benefit of atomoxetine on apathy, clinical trials would more likely include patient- and clinical-rated assessments of behaviour and daily function (as noted in the Discussion, lines 468 – 474).

**Reviewer Comment 2.5**

In Equation 5 - presumably the authors mean N(x^(n),sigma_s^2), not N(s^(n),sigma_s^2) for the likelihood? The likelihood should be a function of the latent parameters (i.e., the parameters described by the prior and posterior).

**Author Reply 2.5**

We thank the Reviewer for bringing this error to our attention, which has been corrected as follows:

“The sensory evidence $s^{(n)}$ is assumed to be the true value of the hidden variable $x^{(n)}$ corrupted by sensory noise, $s^{(n)}=x^{(n)}+\epsilon^{(n)}$, where the noise $\epsilon^{(n)}$ is sampled from a Gaussian distribution with zero mean and variance $\sigma_{s}^{2}$, that is $\epsilon^{(n)}\mathcal{\sim N}(0, \sigma_{s}^{2})$. Thus, the likelihood is assumed to follow a Gaussian distribution with mean $x^{(n)}$ and variance $\sigma_{s}^{2}$. The prior on $x^{(n)}$ is assumed to follow a Gaussian distribution with mean $x_{0}^{(n)}$ and variance $\sigma_{0}^{2}$. Taken together, the posterior of $x^{(n)}$ is proportional to the product of two Gaussian distributions:

$$\begin{aligned} p\left( x^{\left( n \right)} | s^{\left( n \right)} \right)\mathcal{\propto N}\left( s^{\left( n \right)};x^{\left( n \right)},\sigma_{s}^{2} \right)\mathcal{\cdot N}\left( x^{\left( n \right)};x_{0}^{\left( n \right)},\sigma_{0}^{2} \right) \#\left( 5 \right) \end{aligned}$$

(…) We can therefore assume without loss of generality that the optimal estimate equals the posterior mean, which is a precision-weighted sum of the sampled sensory evidence and the prior mean”

**Reviewer 3**

**Reviewer Comment 3.1**

Hezemans et al. present a study of 17 PD and 20 controls. The task was as per a previous study. Participants exerted a force to move a circle by a given distance on the screen, sometimes without feedback. When feedback is absent, they estimate how far they think they pushed the ball. The study shows that motivated people were biased to perceive their exerted force as being closer to the target. PD patients were given atomoxetine, which didn't change performance overall. However patients with MRI evidence of locus coeruleus degeneration showed stronger effects of drug on increasing the bias. The result is timely and novel. Noradrenergic degeneration in PD is of great clinical and scientific interest. Although the sample size is not huge, it appears to be a very clean sample of PD patients, and the analyses are rigorous, Bayesian, and well controlled. An impressive range of control analyses are presented. I had no major criticisms but I have a some comments on the interpretation.

**Author Reply 3.1**

We thank the reviewer for their positive appraisal of our study. We are glad to hear that the Reviewer values our analysis approach, and recognises the importance of noradrenergic degeneration in PD. We appreciate their constructive feedback, which has led us to refine the interpretation of our results.

**Reviewer Comment 3.2**

Comparison with other models of motivation.
The framing of apathy in terms of active inference seems to make qualitatively different predictions to standard econometric theories. I think these should be highlighted, as many readers may not make the connections. In particular, the authors talk about "effort", but in their theory, there seems to be no asymmetry between exerting too much or too little force.

Further, it seems a conceptual leap that a sensory prior that produces action, should translate to a prior over *perception* of action. Can the authors clarify why that should be? In general, I would appreciate more of an interface with existing theories of effort and apathy. Also, are the authors talking about physical or cognitive effort here?

Accordingly an important negative seems to be that PD patients did not exert less force than healthy people, and that noradrenaline did not increase force (and perhaps apathy did not correlate with force either?). Does this inform theories of apathy?

Further, the experimental design (effort x reward) was not analysed as such. Although this aligns with the previous paper, I believe that the reader would be genuinely interested in the effects of those manipulations, especially if they are null results. If the authors do not wish to report these, then are the data at least available?

**Author Reply 3.2**

We thank the Reviewer for their detailed analysis of our theoretical framework, and appreciate their constructive feedback. We have sought to address their specific questions separately below.

**Reviewer Comment 3.2.1**

The framing of apathy in terms of active inference seems to make qualitatively different predictions to standard econometric theories. I think these should be highlighted, as many readers may not make the connections. In particular, the authors talk about "effort", but in their theory, there seems to be no asymmetry between exerting too much or too little force. Further, it seems a conceptual leap that a sensory prior that produces action, should translate to a prior over *perception* of action. Can the authors clarify why that should be? In general, I would appreciate more of an interface with existing theories of effort and apathy.

**Author Reply 3.2.1**

Our study pursued the general principles of meta-cognitive performance beliefs in the context of effortful, goal-directed behaviour, and the modulation of these beliefs by noradrenaline in Parkinson’s disease. We did not aim to provide a comprehensive information processing model of behaviour in the task. We believe that such an approach would be beyond our study and task, unless it were to rely excessively on *ad hoc* solutions and assumptions with regard to the mechanisms of force production and the nature of sensory evidence in our task (as highlighted by the Reviewer). We recognise that this analytic strategy should be explicitly noted – and that its limitations should be discussed – and we have therefore added the following paragraph to the revised Discussion:

We used Bayesian inference as a principled framework to model the computational goal of perceptual inference in the context of effortful, goal-directed behaviour. However, we cannot comment directly on the exact algorithmic underpinnings of Bayesian perceptual inference in our experiment [105]. Following hierarchical models of predictive coding, one could decompose behaviour in our experiment into predictions at multiple levels of abstraction, from low-level proprioceptive predictions for movement to high-level multi-modal, domain-general beliefs [46,106-111]. Future work is needed to address how such a framework can accommodate the various stages of information processing that are thought to be involved in effort- and reward-based decision-making [112-114], including the weighting of action policies [13,15,53], initiating and sustaining an action [115], and evaluating and learning from the action outcomes.

**Reviewer Comment 3.2.2**

Are the authors talking about physical or cognitive effort here?

**Author Reply 3.2.2**

We would characterise our visuomotor task as involving at least physical effort (using the force-pad), and the experimental conditions of low and high effort were designed to manipulate physical effort. However, we cannot wholly exclude the possibility that our task also involved some form of cognitive effort. For example, participants had to learn to apply the right amount of force – neither too much nor too little – which potentially required attentional resources and memory.

In the revised manuscript, we have clarified that the experimental manipulation of effort refers to physical effort:

“For consistency with the original task design in Hezemans et al. [43], the task additionally featured experimental manipulations of effort and reward. Physical effort was manipulated by displaying the target either relatively close to or far from the ball’s starting position, corresponding to 35% or 65% of the participant’s maximum force.”

**Reviewer Comment 3.2.3**

An important negative seems to be that PD patients did not exert less force than healthy people, and that noradrenaline did not increase force (and perhaps apathy did not correlate with force either?). Does this inform theories of apathy?

**Author Reply 3.2.3**

We presume that the Reviewer is referring to our results regarding basic task performance, expressed in terms of the force error – that is, the difference between the force response and the force required to stop the ball perfectly on target. For each subject (and drug condition), we calculated the median force error and interquartile range of force error as summary measures of task accuracy and variability, respectively. The Reviewer correctly points out that there were no significant differences between the Parkinson’s disease group on placebo and the control group, either in terms of the median force error or the interquartile range of force error. Furthermore, among the Parkinson’s disease group there were no significant effects of atomoxetine on either the median force error or interquartile range of force error. Indeed, we designed the visuomotor task with levels of effort that patients could realistically manage throughout the testing session. Given that our Parkinson’s disease cohort was mild-to-moderate, it is not surprising that we saw similar levels of force to their age-matched counterparts. As we were interested in higher-order ‘cognitive’ beliefs about performance and their relationship with apathy and noradrenaline. Lower-level kinematics mechanisms of force production were not the primary focus of the current study, although these too have been studied in terms of active inference in the context of Parkinson’s disease (e.g. Macerollo et al., 2016, 2019).

To address the Reviewer’s question about potential relationships between apathy and force, we entered the median force error and interquartile range of force error as dependent variables in separate ANCOVAs, with the Apathy Scale scores as the between-subjects covariate of interest, and either Group as a between-subjects factor (for analysing the Parkinson’s disease group on placebo and the control group) or Drug as a within-subjects factor (for analysing the Parkinson’s disease group’s full data). Across all models, there were no statistically significant effects of apathy on basic task performance (all *p*’s ≥ .210; all *BF*’s ≤ 1.14).

Answering the question of whether our results inform theories of apathy depends on one’s view of the appropriate level of analysis. The starting point of our research project was to move beyond descriptive models of raw behavioural performance, and instead interrogate the probabilistic beliefs that are thought to *underlie* perception and behaviour (Friston et al., 2014). This enabled us to test specific hypotheses about the role of noradrenaline in mediating individual differences in precision weighting, a latent cognitive parameter that we previously found to be involved in apathy (Hezemans et al., 2020). Thus, the relationship between trait apathy and actual force was not central to the research questions. Neither the presence nor absence of a relationship between actual force and apathy would support our hypothesis.

**Reviewer Comment 3.2.4**

The experimental design (effort x reward) was not analysed as such. Although this aligns with the previous paper, I believe that the reader would be genuinely interested in the effects of those manipulations, especially if they are null results. If the authors do not wish to report these, then are the data at least available?

**Author Reply 3.2.4**

We have written an additional supplementary file that describes the effects of effort and reward on indices of basic task performance as well as prior weighting. See also our reply to Reviewer 1, Comment 1.5. In the revised manuscript, we refer the reader to this supplementary file as follows:

“For consistency with the original study design in Hezemans et al. [43], the task additionally featured experimental manipulations of effort and reward. (…) Following the results of Hezemans et al. [43], showing minimal effects of effort and reward on prior weighting that were not associated with apathy, we report the effects of these conditions on behaviour in the S4 text.”

**Reviewer Comment 3.3**

Intro - NA is presented as definitively signalling uncertainty. While there are reasonable arguments for this, I think some caution is wise in some of the stronger statements eg L88.

**Author Reply 3.3**

We agree with the Reviewer that the literature around the role of noradrenaline is nuanced, and we had not conveyed this in certain lines in the introduction. We have revised lines 88 and 113 in the Introduction as follows:

“A growing body of work suggests that noradrenaline signals the uncertainty of an individual’s internal model of their environment. (…) Crucially, precision weighting has been associated with neurotransmitter systems that include noradrenaline [50-53]”

**Reviewer Comment 3.4**

I think "optimal" is used in an unusual way (page 8) - the authors write about the optimal integration, but this seems odd since the optimal prior actually has zero-weight (no precision). This is because the sensory input is not biased away from the target (I assume?) -- so it should always be "better" to use raw sensory information rather than integrate it with the target. People of course do mix the prior with evidence, but it is confusing to call this optimal.

**Author Reply 3.4**

We agree. In the general setting of perceptual judgements, the Bayesian integration of the likelihood with a prior is optimal, in the sense that the sensory evidence is “regularised” by the prior to reduce the influence of noise. We used this context to introduce our modelling approach in the Materials and Methods (subsection Bayesian Modelling of Performance Estimates). In many behavioural experiments, the stimulus is presented unambiguously (i.e., the likelihood is extremely precise), and hence the influence of the prior on the perceptual estimate practically vanishes. In our experiment however, participants were not shown the ball’s trajectory during estimation trials, and therefore their sensory evidence was presumably relatively ambiguous (imprecise). As a result, participants’ inferences relied heavily on their prior belief, yielding estimates that are objectively inaccurate (i.e., drawn towards the target and away from the true final ball position). However, we emphasise that these inaccurate estimates can nevertheless be regarded as the result of optimal (Bayesian) inference. That is, we frame the participant’s maladaptive behaviour as the result of optimal inference that is based on *their particular generative model of the world* (Schwartenbeck et al., 2015).

As the Reviewer points out, in the context of our particular experiment, one could argue that it would have been adaptive for participants to maintain a flat prior over the true final ball position, so that the perceptual estimates would be centred on the sensory evidence. However, the results of the current study, as well as our previous work (Wolpe et al., 2014, 2015; Hezemans et al., 2020), suggest that participants typically do not make use of such a flat prior, and instead use a relatively precise prior that is centred on the target (i.e., a strong prior belief of successful performance). Overly precise priors may reduce learning rates, but as discussed in Wolpe et al. (2014), there is a potential advantage in terms of sustaining motivation and regularising behaviour in a volatile environment.

To address the Reviewer’s concerns, the revised description of the Bayesian estimation framework (Results section) no longer refers to perceptual estimates as “optimal”:

“We next examined participants’ estimates of their own performance, to infer the relative weight afforded to prior beliefs in the perception of action outcomes. Following Bayes’ rule, participants are assumed to combine their prior belief, centred on the target, with trial-wise sensory evidence, centred on the (hidden) true final ball position, to estimate the ball’s final position (Fig 1B). Assuming that the prior and sensory evidence are represented as Gaussian distributions with unknown variances $\sigma_{\mathrm{prior}}^{2}$ and $\sigma_{\mathrm{evidence}}^{2}$, the posterior estimate for a given trial $n$ ($x_{\mathrm{estimate}}^{(n)}$) is a precision-weighted sum of the target position $x_{\mathrm{target}}^{(n)}$ and the true final ball position $x_{\mathrm{ball}}^{(n)}$”

We made analogous changes to the S5 text:

“For a given estimation trial $n$, the posterior estimate of the final ball position is a precision-weighted combination of the prior (centred on the target position) and the likelihood (centred on the simulated true final ball position)”

**Reviewer Comment 3.5**

I would prefer if some of the task details in Materials & Methods appeared up in the main text e.g. how the force determined the distance moved, the proportion of trials.

**Author Reply 3.5**

We agree and have revised the paragraph in question as follows:

“The visuomotor task involved effortful, goal-directed behaviour, and was designed to estimate prior weighting, i.e. the relative precision of prior beliefs and sensory evidence in the perception of outcomes. (…) The force exerted on the sensor (relative to the participant’s maximum force, measured separately) determined the initial velocity of the ball. Since the deceleration of the ball was constant, the force response (i.e., initial velocity) directly determined the ball’s final position. The difference between the ball’s final position and the target constituted the performance error. For 40 out of the total 120 trials, the ball’s trajectory was not shown, and participants were asked to estimate where the ball would have stopped, indicating their response on a grid of 12 response options (Fig 1A; see Materials and Methods for details).”

The Reviewer’s comment also encouraged us to refine our description of the visuomotor task in the Materials and Methods:

“The force response was defined as the mean force exerted from 1.5 to 2.5 seconds, divided by the participant’s maximum force (estimated separately, see below). The initial velocity of the ball increased monotonically with the force response. The deceleration of the ball was kept constant. Thus, the relative force exerted on the sensor (i.e., the initial velocity) directly determined the ball’s final position.”

**Reviewer Comment 3.6**

Minor comment: L225: a healthy

**Author Reply 3.6**

We thank the Reviewer for spotting this typo. The article “a” has been removed:

“This corroborates the negative relationship between prior weighting and trait apathy in ~~a~~ healthy young adults”

**Reviewer Comment 3.7**

*Have the authors made all data and (if applicable) computational code underlying the findings in their manuscript fully available?* Reviewer 3: No: Did not see it in text - maybe I missed it.

**Author Reply 3.7**

Yes. We have prepared a repository on the Open Science Framework containing all code and data that is necessary to reproduce the manuscript’s results. However, the Reviewer rightly points out that the submitted manuscript did not contain functioning links to this repository.

Our current policy is to release a manuscript’s code and materials upon acceptance for publication. We have now created a confidential “view-only” link to the Open Science Framework repository (<https://osf.io/mry4f/?view_only=9201a50a0b094c45bbe8cacac44a5c41>). If accepted for publication, we will replace this temporary link with a final (public) link to the repository, as part of the proofing process.

**References**

Aston-Jones, G., & Cohen, J. D. (2005). An integrative theory of locus coeruleus-norepinephrine function: adaptive gain and optimal performance. *Annual Review of Neuroscience*, *28*, 403-450.

Bymaster, F. P., Katner, J. S., Nelson, D. L., Hemrick-Luecke, S. K., Threlkeld, P. G., Heiligenstein, J. H., ... & Perry, K. W. (2002). Atomoxetine increases extracellular levels of norepinephrine and dopamine in prefrontal cortex of rat: a potential mechanism for efficacy in attention deficit/hyperactivity disorder. *Neuropsychopharmacology*, *27*(5), 699-711.

Chong, T. T. J., Bonnelle, V., Manohar, S., Veromann, K. R., Muhammed, K., Tofaris, G. K., ... & Husain, M. (2015). Dopamine enhances willingness to exert effort for reward in Parkinson's disease. *Cortex*, *69*, 40-46.

Cools, R. (2019). Chemistry of the adaptive mind: lessons from dopamine. *Neuron*, *104*(1), 113-131.

Cumming, G. (2014). The new statistics: Why and how. *Psychological science*, *25*(1), 7-29.

Dayan, P., & Yu, A. J. (2006). Phasic norepinephrine: a neural interrupt signal for unexpected events. *Network: Computation in Neural Systems*, *17*(4), 335-350.

Devoto, P., & Flore, G. (2006). On the origin of cortical dopamine: is it a co-transmitter in noradrenergic neurons?. *Current neuropharmacology*, *4*(2), 115-125.

Eldar, E., Cohen, J. D., & Niv, Y. (2013). The effects of neural gain on attention and learning. *Nature Neuroscience*, *16*(8), 1146-1153.

Friston, K. J., Stephan, K. E., Montague, R., & Dolan, R. J. (2014). Computational psychiatry: the brain as a phantastic organ. *The Lancet Psychiatry*, *1*(2), 148-158.

Gottlieb, J., & Oudeyer, P. Y. (2018). Towards a neuroscience of active sampling and curiosity. *Nature Reviews Neuroscience*, *19*(12), 758-770.

Hauser, T. U., Allen, M., Purg, N., Moutoussis, M., Rees, G., & Dolan, R. J. (2017). Noradrenaline blockade specifically enhances metacognitive performance. *Elife*, *6*, e24901.

Hezemans, F. H., Wolpe, N., & Rowe, J. B. (2020). Apathy is associated with reduced precision of prior beliefs about action outcomes. *Journal of Experimental Psychology: General*, *149*(9), 1767.

Hommel, B., Müsseler, J., Aschersleben, G., & Prinz, W. (2001). The theory of event coding (TEC): A framework for perception and action planning. *Behavioral and brain sciences*, *24*(5), 849-878.

Jepma, M., Murphy, P. R., Nassar, M. R., Rangel-Gomez, M., Meeter, M., & Nieuwenhuis, S. (2016). Catecholaminergic regulation of learning rate in a dynamic environment. *PLoS computational biology*, *12*(10), e1005171.

Kehagia, A. A., Housden, C. R., Regenthal, R., Barker, R. A., Müller, U., Rowe, J., ... & Robbins, T. W. (2014). Targeting impulsivity in Parkinson’s disease using atomoxetine. *Brain*, *137*(7), 1986-1997.

Lawson, R. P., Bisby, J., Nord, C. L., Burgess, N., & Rees, G. (2021). The computational, pharmacological, and physiological determinants of sensory learning under uncertainty. *Current Biology*, *31*(1), 163-172.

Macerollo, A., Chen, J. C., Korlipara, P., Foltynie, T., Rothwell, J., Edwards, M. J., & Kilner, J. M. (2016). Dopaminergic treatment modulates sensory attenuation at the onset of the movement in Parkinson's disease: A test of a new framework for bradykinesia. *Movement Disorders*, *31*(1), 143-146.

Macerollo, A., Limousin, P., Korlipara, P., Foltynie, T., Edwards, M. J., & Kilner, J. (2019). Dopaminergic Modulation of Sensory Attenuation in Parkinson's Disease: Is There an Underlying Modulation of Beta Power?. *Frontiers in Neurology*, 1001.

Mirza, M. B., Adams, R. A., Mathys, C., & Friston, K. J. (2018). Human visual exploration reduces uncertainty about the sensed world. *PloS one*, *13*(1), e0190429.

Nair, A. B., & Jacob, S. (2016). A simple practice guide for dose conversion between animals and human. *Journal of basic and clinical pharmacy*, *7*(2), 27.

Nassar, M. R., Rumsey, K. M., Wilson, R. C., Parikh, K., Heasly, B., & Gold, J. I. (2012). Rational regulation of learning dynamics by pupil-linked arousal systems. *Nature Neuroscience*, *15*(7), 1040-1046.

O’Callaghan, C., Hezemans, F. H., Ye, R., Rua, C., Jones, P. S., Murley, A. G., ... & Rowe, J. B. (2021). Locus coeruleus integrity and the effect of atomoxetine on response inhibition in Parkinson’s disease. *Brain*, *144*(8), 2513-2526.

Parr, T., Sajid, N., Da Costa, L., Mirza, M. B., & Friston, K. J. (2021). Generative models for active vision. *Frontiers in Neurorobotics*, *15*, 34.

Ramstead, M. J., Kirchhoff, M. D., & Friston, K. J. (2020). A tale of two densities: Active inference is enactive inference. *Adaptive Behavior*, *28*(4), 225-239.

Rae, C. L., Nombela, C., Rodríguez, P. V., Ye, Z., Hughes, L. E., Jones, P. S., ... & Rowe, J. B. (2016). Atomoxetine restores the response inhibition network in Parkinson’s disease. *Brain*, *139*(8), 2235-2248.

Robbins, T. W. (2000). Chemical neuromodulation of frontal-executive functions in humans and other animals. *Experimental brain research*, *133*(1), 130-138.

Sales, A. C., Friston, K. J., Jones, M. W., Pickering, A. E., & Moran, R. J. (2019). Locus Coeruleus tracking of prediction errors optimises cognitive flexibility: An Active Inference model. *PLoS computational biology*, *15*(1), e1006267.

Schwartenbeck, P., FitzGerald, T. H., Mathys, C., Dolan, R., Wurst, F., Kronbichler, M., & Friston, K. (2015). Optimal inference with suboptimal models: addiction and active Bayesian inference. *Medical hypotheses*, *84*(2), 109-117.

Seamans, J. K., & Yang, C. R. (2004). The principal features and mechanisms of dopamine modulation in the prefrontal cortex. *Progress in neurobiology*, *74*(1), 1-58.

Sesack, S. R., Hawrylak, V. A., Matus, C., Guido, M. A., & Levey, A. I. (1998). Dopamine axon varicosities in the prelimbic division of the rat prefrontal cortex exhibit sparse immunoreactivity for the dopamine transporter. *Journal of Neuroscience*, *18*(7), 2697-2708.

Swanson, C. J., Perry, K. W., Koch-Krueger, S., Katner, J., Svensson, K. A., & Bymaster, F. P. (2006). Effect of the attention deficit/hyperactivity disorder drug atomoxetine on extracellular concentrations of norepinephrine and dopamine in several brain regions of the rat. *Neuropharmacology*, *50*(6), 755-760.

Umehara, M., Ago, Y., Fujita, K., Hiramatsu, N., Takuma, K., & Matsuda, T. (2013). Effects of serotonin–norepinephrine reuptake inhibitors on locomotion and prefrontal monoamine release in spontaneously hypertensive rats. *European journal of pharmacology*, *702*(1-3), 250-257.

Usher, M., Cohen, J. D., Servan-Schreiber, D., Rajkowski, J., & Aston-Jones, G. (1999). The role of locus coeruleus in the regulation of cognitive performance. *Science*, *283*(5401), 549-554.

Wayment, H. K., Schenk, J. O., & Sorg, B. A. (2001). Characterization of extracellular dopamine clearance in the medial prefrontal cortex: role of monoamine uptake and monoamine oxidase inhibition. *Journal of Neuroscience*, *21*(1), 35-44.

Wolpe, N., Nombela, C., & Rowe, J. B. (2015). Dopaminergic modulation of positive expectations for goal-directed action: evidence from Parkinson’s disease. *Frontiers in psychology*, *6*, 1514.

Wolpe, N., Wolpert, D. M., & Rowe, J. B. (2014). Seeing what you want to see: priors for one's own actions represent exaggerated expectations of success. *Frontiers in Behavioral Neuroscience*, *8*, 232.

Ye, Z., Altena, E., Nombela, C., Housden, C. R., Maxwell, H., Rittman, T., ... & Rowe, J. B. (2015). Improving response inhibition in Parkinson’s disease with atomoxetine. *Biological Psychiatry*, *77*(8), 740-748.
